# Supplementary material for: Optimization of 5′ Untranslated Region of Modified mRNA for Use in Cardiac or Hepatic Ischemic Injury
Source: Mol Ther Methods Clin Dev. 2020 Mar 31;17:622–33. doi: 10.1016/j.omtm.2020.03.019 (PMC7150433; doi:10.1016/j.omtm.2020.03.019)
Supplement: Document S1. Figures S1–S5 and Table S1 [file mmc1.pdf]

OMTM, Volume *17*

Supplemental Information

Optimization of 5′ Untranslated Region of Modified mRNA for Use in Cardiac or Hepatic Ischemic Injury

Nishat Sultana, Yoav Hadas, Mohammad Tofael Kabir Sharkar, Keerat Kaur, Ajit Magadum, Ann Anu Kurian, Nadia Hossain, Bremy Alburquerque, Sakib Ahmed, Elena Chepurko, and Lior Zangi

Supplementary Information

Table of Contents

1) Supplementary Figures

2) Supplementary Table 1

3) Author Contributions

1. Supplementary figures

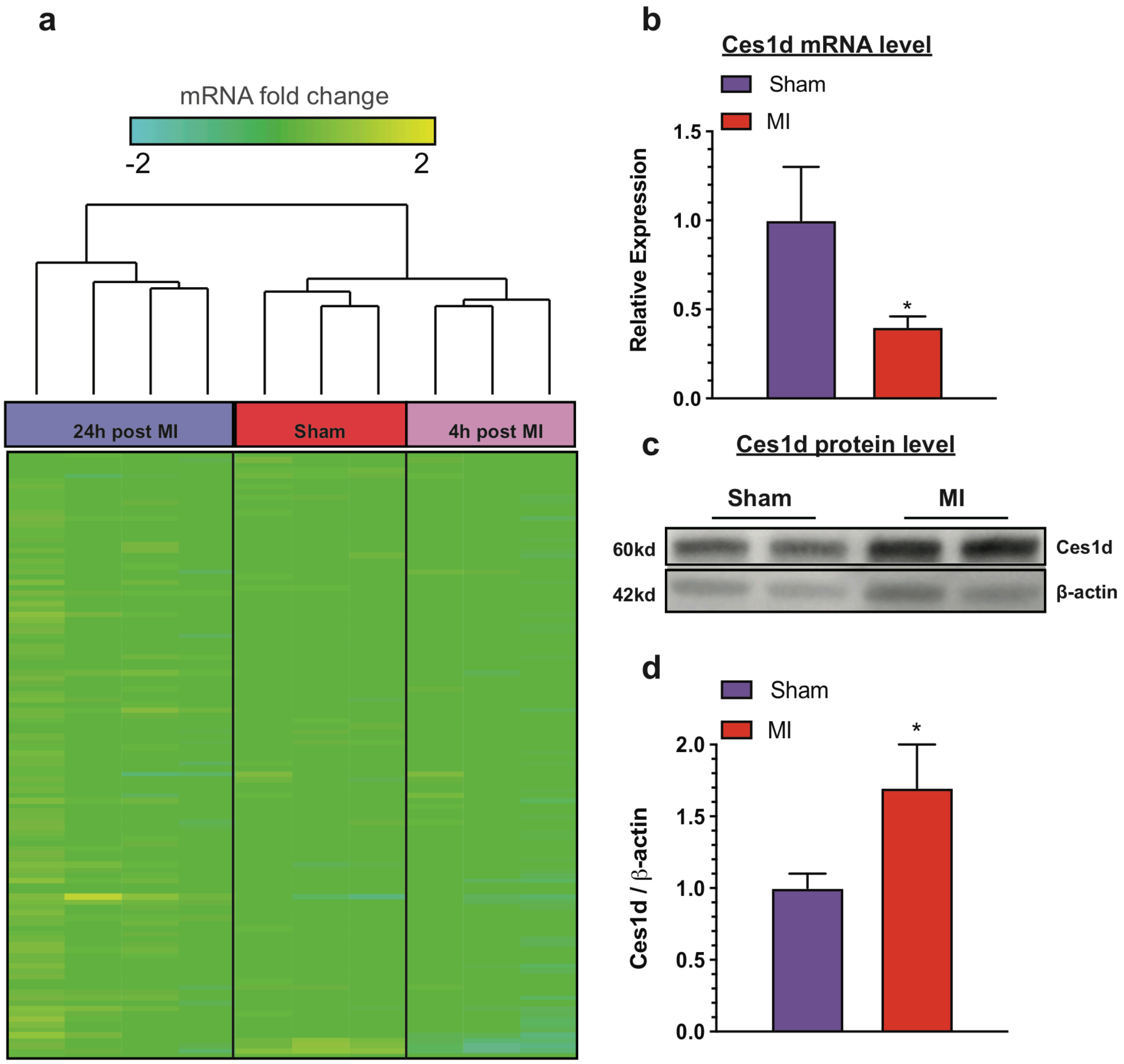

Supplementary Figure 1: RNAseq experimental groups are clusters together in hierarchical clustering dendrogram and Ces1d qPCR and western blot confirm RNAseq and proteomic expression results. a. Hierarchical clustering dendrogram of 14,000 genes that was sequenced for transcriptomic analysis from ischemic or non-ischimic heart tissues (n=10 total, Sham n=3, 4 hours post MI n=3 or 24 hours post MI n=4). **b.** qPCR to evaluate Ces1d expression was done for RNA samples taken 24 hours from hearts undergo Sham or MI surgery (n=3). **c.** Representative image of western blot analysis done with anti Ces1d antibody to evaluate protein samples taken 24 hours from hearts undergo Sham or MI surgery. **d.** Quantification of the experiments shown in c (n=2). Unpaired two-tailed t-test for b&d. *, $P<0.05$.

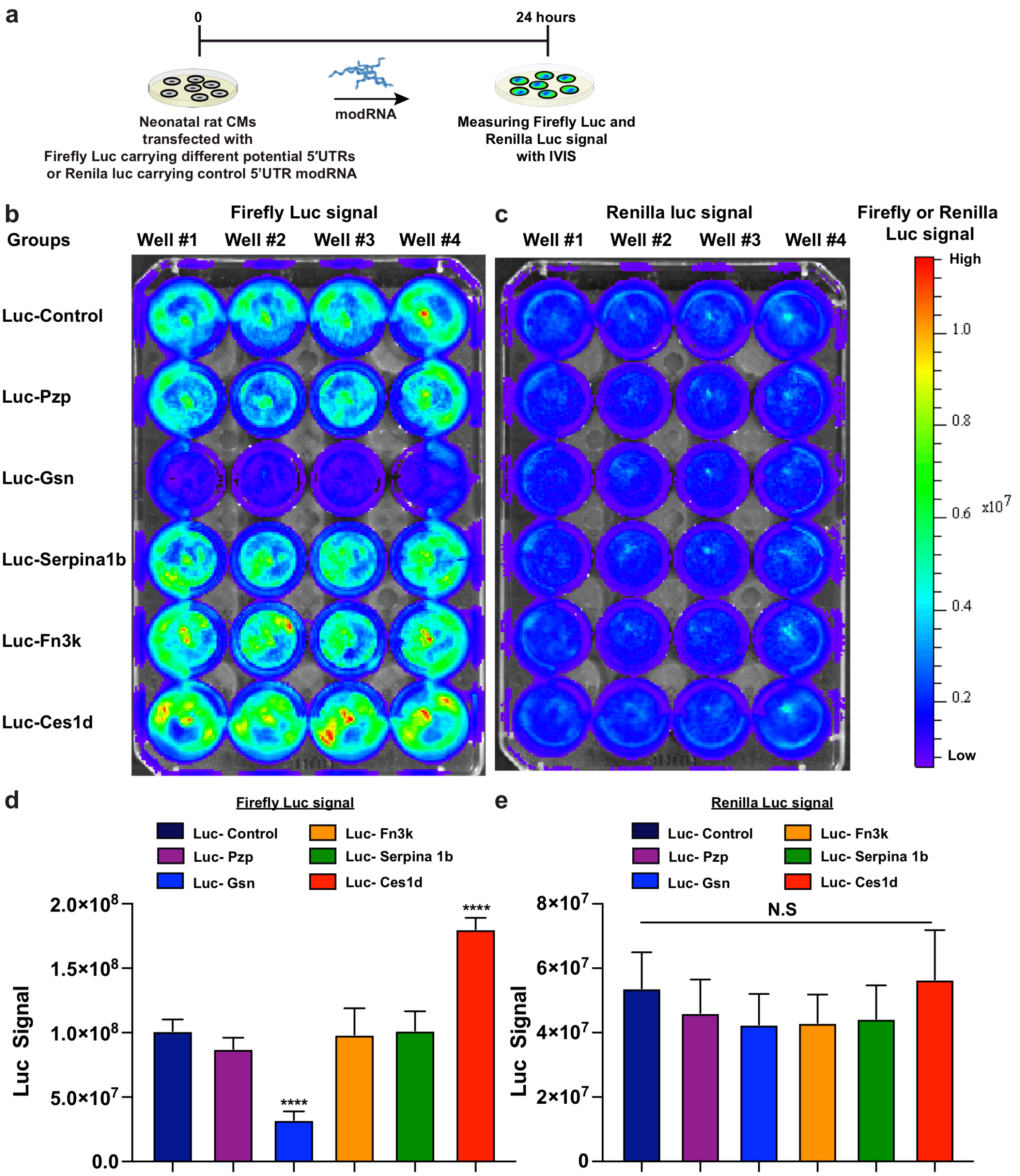

Supplementary Figure 2. Representative image from IVIS analysis 24 hours post transfection with Luc modRNA carrying different potential 5′UTRs and Renilla modRNA (transfection control) in neonatal rat CMs.

a. Experimental plan to evaluate the translation efficiency of different potential 5′UTRs in neonatal rat CMs using Luc modRNA and IVIS analysis.

b&c. Representative IVIS analysis (**b** for luciferase (Luc) signal and **c** for renilla signal) image of the results that was received in the experiment that was described in a. **d&e.** Quantification of the IVIS analysis that describe in b&c, respectively. One-way ANOVA, Tukey's Multiple Comparison Test for d&e.
****, $P<0.0001$, ***, $P<0.001$, N.S, Not Significant.

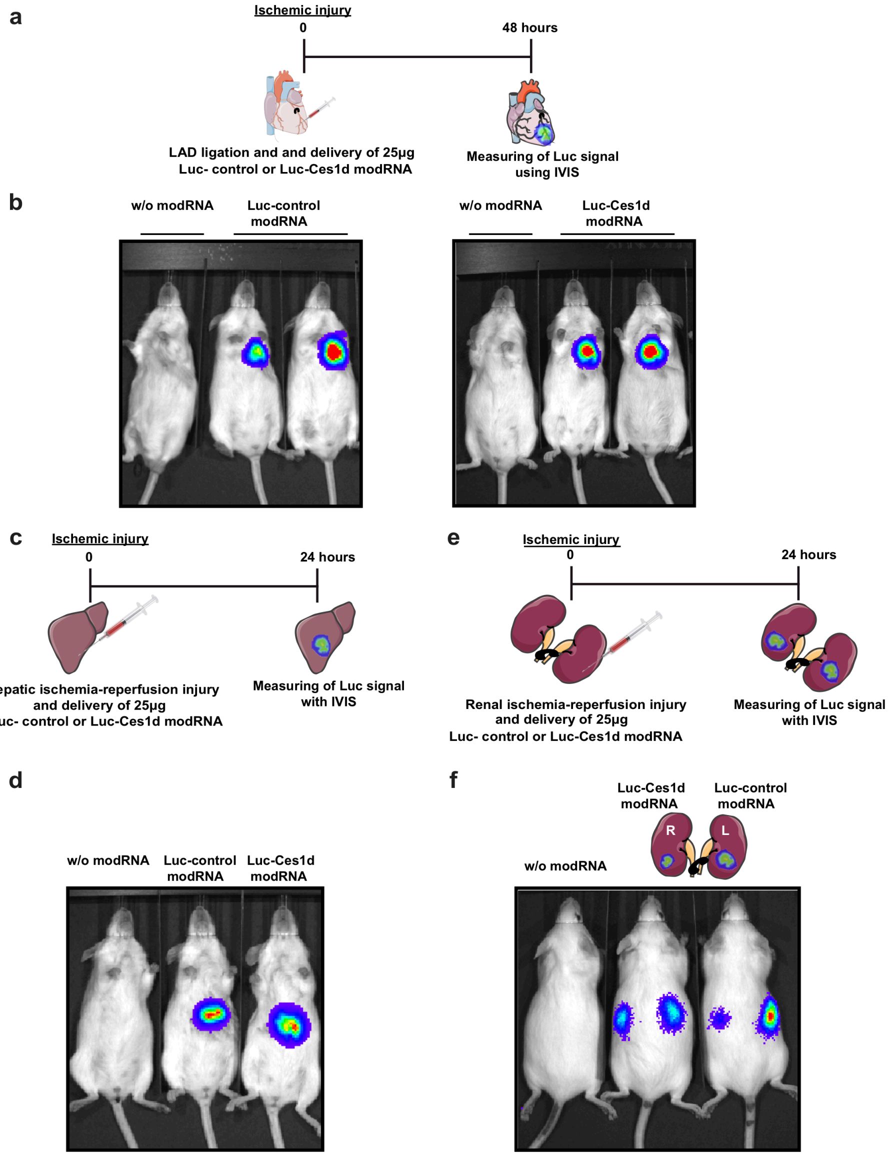

Supplementary Figure 3. Representative images of Luc modRNA translation efficiency when carrying 5′UTR of Ces1d or artificial (control) 5’UTR in ischemic heart, kidney or liver mouse models.

a. Experimental plan to evaluate the translation efficiency of Luc modRNA carrying 5′UTR of Ces1d or artificial (control) 5’UTR in ischemic heart mouse model. **b.** Representative IVIS images of the results that was received in the experiment that was described in a. **c.** Experimental plan to evaluate the translation efficiency of Luc modRNA carrying 5′UTR of Ces1d or artificial (control) 5’UTR in ischemic liver mouse model. **d.** Representative IVIS image of the results that was received in the experiment that was described in c. **e.** Experimental plan to evaluate the translation efficiency of Luc modRNA carrying 5′UTR of Ces1d or artificial (control) 5’UTR in ischemic kidney mouse model. **f.** Representative IVIS image of the results that was received in the experiment that was described in e.

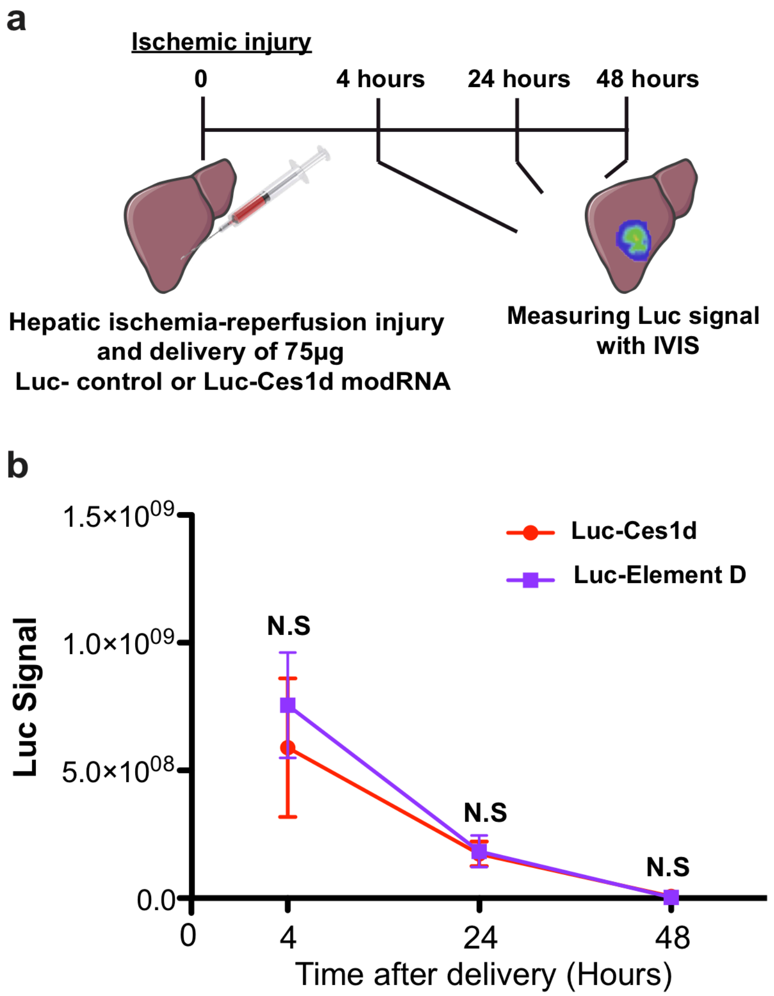

Supplementary Figure 4. Element D in 5'UTR of Ces1d hasn't increased significantly mRNA translation over full length Ces1d 5'UTR in liver ischemic mouse model.

a. Experimental plan to evaluate the translation efficiency of Luc modRNA carrying full length 5′UTR of Ces1d or only Element D in ischemic liver mouse model. **b.** Quantification of the experiments that was described in a (n=4). Two-way ANOVA, Tukey's Multiple Comparison Test. N.S, Not Significant.

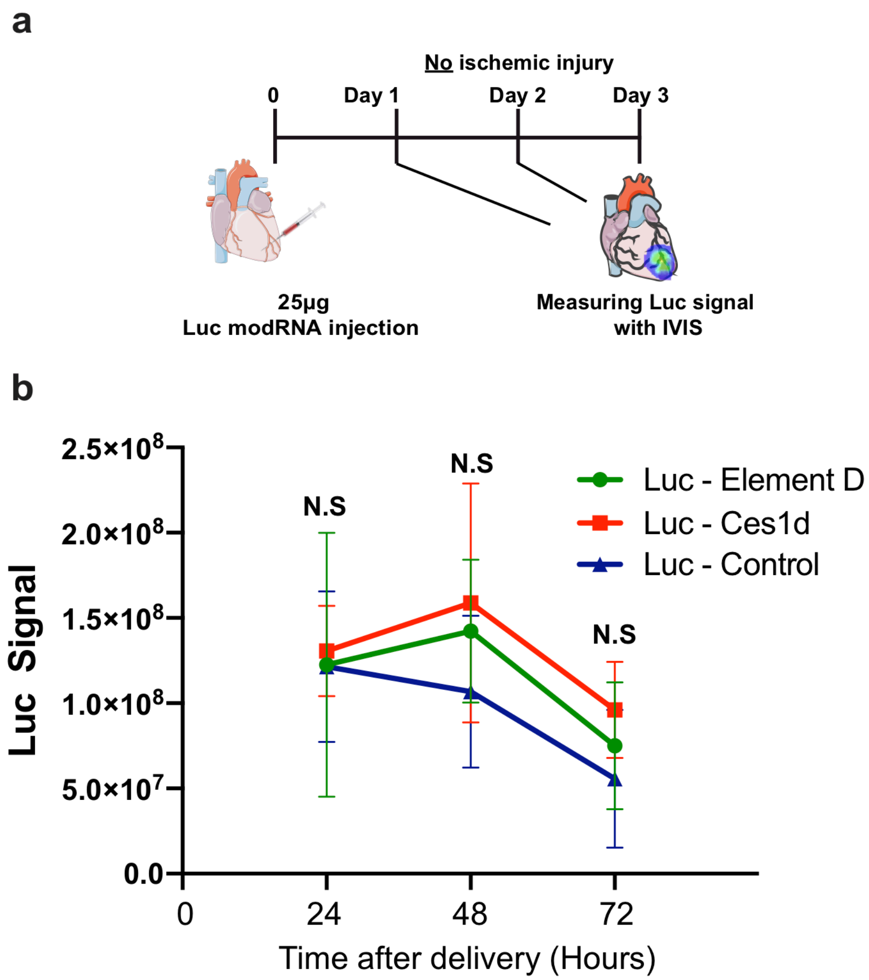

Supplementary Figure 5. Element D in 5'UTR of Ces1d hasn't increased significantly mRNA translation over full length Ces1d or control 5'UTR in heart non-ischemic mouse model.

a. Experimental plan to evaluate the translation efficiency of Luc modRNA carrying full length Ces1d or control 5'UTR in heart non-ischemic mouse model.
b. Quantification of the experiments that was described in a (n=6). Two-way ANOVA, Tukey's Multiple Comparison Test. N.S, Not Significant.

2. Supplementary Table

Supplementary Table 1: 5’UTR sequence of the 5 genes with the shortest 5’UTR length from the list in Fig. 1f&g.

Gene name	5’UTR Sequence
Gsn	gctagggcgggatgggacggccggttacttaaaggttggg gcgaccaagggtccgcggccgcagcctgggtcctcaccgt cgcc
Pzp	caaggatcagagttcgggggctgagggctcagacgttcttctctgccctc tccacc
Serpina 1b	atatccccccttggctcccactgcttaaatacagactaggacagggctctg tctcctcagcctcggtcaccacccagctctgggacagcaagctgaaa
Fn3k	tgcgtcacctgaccgcattctgcacctcaactctcc
Ces1d	aggaggcgggtcccctggtccacaacagaagcattgctaaagcagcagat agctcagagacccacagagcccttgtccttccaca

3. Author Contributions

N.S. designed the study, carried out most of the experiments, analyzed most of the data and wrote the manuscript, Y.H. designed, carried out and analyzed the RNAseq and proteomics experiments and revised the manuscript, M.T.K.S prepared modRNAs and performed experiments, K.K. performed experiments and revised the manuscript, A.M. revised the manuscript, N.H., B.A and S.A., perform experiment and help analyzing the data, E.C. carried out all surgery and modRNA injections in mouse models, L.Z. designed the experiments, analyzed data and wrote the manuscript .
