# Supplementary material for: Optimization of 5′ Untranslated Region of Modified mRNA for Use in Cardiac or Hepatic Ischemic Injury
Source: Mol Ther Methods Clin Dev. 2020 Mar 31;17:622–33. doi: 10.1016/j.omtm.2020.03.019 (PMC7150433; doi:10.1016/j.omtm.2020.03.019)
Supplement: Document S2. Article plus Supplemental Information [file mmc2.pdf]

Molecular Therapy
Methods & Clinical Development
Original Article

Optimization of 5′ Untranslated Region of Modified mRNA for Use in Cardiac or Hepatic Ischemic Injury

Nishat Sultana,[1,2,3,4] Yoav Hadas,[1,2,3,4] Mohammad Tofael Kabir Sharkar,[1,2,3] Keerat Kaur,[1,2,3] Ajit Magadum,[1,2,3] Ann Anu Kurian,[1,2,3] Nadia Hossain,[1,2,3] Bremy Alburquerque,[1,2,3] Sakib Ahmed,[1,2,3] Elena Chepurko,[1,2,3] and Lior Zangi[1,2,3]

[1]Cardiovascular Research Center, Icahn School of Medicine at Mount Sinai, New York, NY 10029, USA; [2]Department of Genetics and Genomic Sciences, Icahn School of Medicine at Mount Sinai, New York, NY 10029, USA; [3]Black Family Stem Cell Institute, Icahn School of Medicine at Mount Sinai, New York, NY 10029, USA

Modified mRNA (modRNA) is a gene-delivery platform for transiently introducing a single gene or several genes of interest to different cell types and tissues. modRNA is considered to be a safe vector for gene transfer, as it negligibly activates the innate immune system and does not compromise the genome integrity. The use of modRNA in basic and translational science is rising, due to the clinical potential of modRNA. We are currently using modRNA to induce cardiac regeneration post-ischemic injury. Major obstacles in using modRNA for cardiac ischemic disease include the need for the direct and single administration of modRNA to the heart and the inefficient translation of modRNA due to its short half-life. Modulation of the 5′ untranslated region (5′ UTR) to enhance translation efficiency in ischemic cardiac disease has great value, as it can reduce the amount of modRNA needed per delivery and will achieve higher and longer protein production post-single delivery. Here, we identified that 5′ UTR, from the fatty acid metabolism gene carboxylesterase 1D (Ces1d), enhanced the translation of firefly luciferase (Luc) modRNA by 2-fold in the heart post-myocardial infarction (MI). Moreover, we identified, in the Ces1d, a specific RNA element (element D) that is responsible for the improvement of modRNA translation and leads to a 2.5-fold translation increment over Luc modRNA carrying artificial 5′ UTR, post-MI. Importantly, we were able to show that 5′ UTR Ces1d also enhances modRNA translation in the liver, but not in the kidney, post-ischemic injury, indicating that Ces1d 5′ UTR and element D may play a wider role in translation of protein under an ischemic condition.

INTRODUCTION

Ischemic heart disease is the leading cause of death for both men and women in the United States, killing about 610,000 people per year.[1,2] Many scientists around the world are studying different approaches to induce cardiac regeneration in hope to unravel new treatments that can improve cardiac function post-ischemic injury. Genetic medicine is one of the avenues of research to treat a failing heart post-myocardial infarction (MI).[3] In this research, we aimed to adjust gene expression in the heart using viral vectors, small molecules, or an RNA-based approach to promote cardiac protection and cardiovascular or cardiac regeneration in ischemic cardiac disease. Modified messenger RNA (modRNA) is a novel gene therapy platform that can be used to alter the levels of proteins in mammalian cells and tissues[4,5] and to treat heart diseases.[6–8] The concept of therapeutically altering mRNA expression has great potential in the treatment of human diseases.[9] To date, several therapeutic approaches using small interfering RNA (siRNA) and antisense oligonucleotides have been successfully tested to reduce mRNA levels in the cells.[10,11] Yet, the upregulation of proteins in tissues is challenging, mostly to the high amount of mRNA needed to treat human tissue. The supply of a large amount of mRNA *in vivo* can subsequently elicit a higher immune response. Preclinical studies with modRNA showed that due to the transient expression of modRNA (gene expression return to baseline is a few days), modRNA will be needed, both in direct or intravenous delivery methods, to be redelivered to achieve higher gene expression.[6,12–15]

The expression of genes is controlled intricately at the post-transcriptional level.[16] The level of an individual mRNA type inside of a cell does not ensure the synthesis of comparable amounts of respective proteins,[17] both positive and negative modulators that influence translation and maintain certain levels of protein. Eukaryotic gene translation is regulated in the translation level by several components, including 5′ untranslated region (UTR),[18–20] 3′ UTR,[21–23] poly(A) tail,[24–27] cap structure,[28–31] etc. There are multiple regulatory elements within the untranslated regions (UTRs) of the mRNA, which are critical for the stability and translation of mRNA into protein.[32,33] Moreover, the length and secondary structure of 5′ UTR and mutations within the 5′ UTR have been reported to be associated with certain human diseases.[34] The 5′ UTR plays a significant role in the

Received 5 February 2020; accepted 23 March 2020;
https://doi.org/10.1016/j.omtm.2020.03.019.

[4]These authors contributed equally to this work.

Correspondence: Lior Zangi, Icahn School of Medicine at Mount Sinai, New York, NY 10029, USA.
E-mail: lior.zangi@mssm.edu

 © 2020 The Author(s).
This is an open access article under the CC BY license (http://creativecommons.org/licenses/by/4.0/).

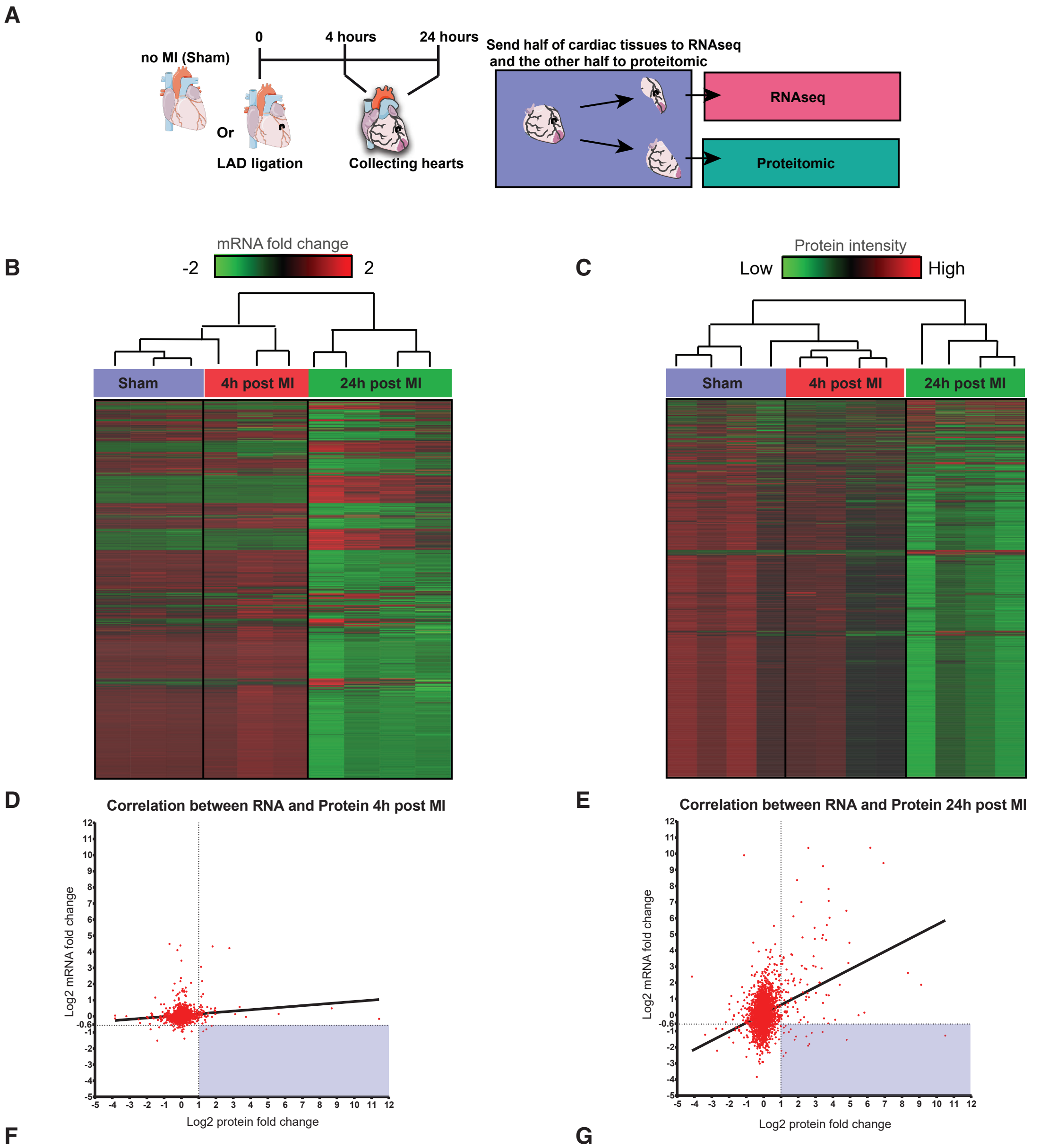

F

#	Gene ID	protein fold change (MI vs Sham)	RNA fold change (MI vs Sham)	5' UTR length
1	Pygl	3.36	0.66	100
2	Serpina 1b	5.93	0.57	97
3	Frmd5	2.4	0.52	226

G

#	Gene ID	protein fold change (MI vs Sham)	RNA fold change (MI vs Sham)	5' UTR length
1	Smarca2	1446.68	0.41	110
2	Vtn	27.89	0.34	230
3	Gsn	10.62	0.48	84
4	Pzp	6.86	0.46	56
5	Igsf10	6.24	0.29	568
6	Serpina1b	5.93	0.27	97
7	Fbln1	5.53	0.40	126
8	Flad1	3.66	0.40	216
9	Ppip5k2	3.45	0.35	503
10	Scrn2	2.96	0.54	104
11	Etfrf1	2.75	0.48	386
12	Frmd5	2.42	0.20	226
13	Itm2b	2.41	0.63	145
14	Tmem201	2.24	0.47	121
15	Clpx	2.24	0.58	227
16	Fn3k	2.21	0.17	36
17	Ces1d	2.04	0.22	85
18	Hmgn2	2.01	0.61	182

(legend on next page)

regulation of translational efficiency by helping the ribosome to bind the messenger RNA (mRNA) in the proximity of the start codon and thus, is a main contributor to the cellular proteome.[35] Additionally, certain RNA elements within the 5′ UTR may change its secondary structure (e.g., internal ribosome entry sites [IRESs], upstream start codon (AUG)s, or upstream open reading frames [uORFs]) and can be an important contributor of the entire translation rate.[36,37] Besides, 5′ UTRs can contain sequence elements that can function as binding sites for regulatory proteins.[33]

Until this date, modRNA, which had been used preclinically in cardiac research using artificial 5′ UTR (36 nt), was first described by Warren et al.[38]

Asrani et al.[39] has used *in vitro* screening for optimization mRNA 5′ UTR to improve expression of arginase 1 (ARG1). They showed that plasmid-based screening methods do not correlate with protein expression driven by exogenously expressed mRNA and that improved 5′ UTR but not 3′ UTR appears to be the key driver in protein expression for exogenously delivered mRNA.

We recently optimized the synthesis[5] and the delivery[4] of modRNA for cardiac delivery use. In the present study, we compared transcriptomic and proteomic analysis of similar tissues to identify, *in vivo*, a potential 5′ UTR and the elements within 5′ UTR that can enhance the translation post-cardiac and hepatic ischemic injury condition.

RESULTS

Characterizing the Ischemic Heart Transcriptome and Proteome

To characterize the dynamics of heart left-ventricle (LV) transcriptome and proteome post-MI, we analyzed changes in genes expression and protein level in the LV of mice, 4 and 24 h post-MI and compared it to LV from sham-operated mice (Figure 1A). In total, we detected 14,552 genes and 2,397 proteins in our samples. With the comparison of the two datasets, we found 2,272 genes with corresponding proteins. Out of all genes with corresponding proteins, 239 genes and 120 proteins were differentially expressed (q value < 0.05), 4 h post-MI. 24 h post-MI, 1,702 genes and 272 proteins were differentially expressed. Hierarchical clustering dendrogram of gene expression (Figures 1B and S1A) and protein levels (Figure 1C) shows that in both cases, the experimental groups are clustered together, demonstrating significant differences in the transcriptome and proteome post-MI. Pearson correlation analysis revealed a positive correlation between changes in gene expression and protein expression, both 4 h post-MI (r squared = 0.02; Figure 1D) and 24 h post-MI (r squared = 0.13; Figure 1E). Whereas we found a significant correlation between the changes in gene expression and protein levels in the ischemic heart post-MI and in our search for a 5′ UTR element that may elevate translation of modRNA in the heart post-MI, we were interested in identifying genes with a significant noncorrelation relationship between mRNA and protein expression. Therefore, we screened for genes that encode for proteins with elevated levels in 4 or 24 h post-MI (fold change > 2) and mRNA downregulated at 4 or 24 h post-MI (fold change < 0.64) and 5′ UTR shorter than 100 bp. We were able to identify 3 genes in 4 h (Figure 1F) and 18 genes in 24 h (Figure 1G) that displayed high protein expression accompanied with lower or unchanged mRNA level, 24 h post-MI, compared to sham hearts. We identify 5 genes (gelsolin [Gsn], pregnancy-zone protein [Pzp], Serpina, fructosamine 3 kinase [Fn3k], and carboxylesterase 1D [Ces1d], marked in yellow, in Figures 1F and 1G) that had the shortest 5′ UTR among those 19 genes (as FERM domain containing 5 [Frmd5] and Serpina 1b are present in both our 4- and 24-h screen results), with an upregulated protein expression not related to their mRNA expression post-MI. In addition, we validate Ces1d expression results using qPCR and western blot, showing similar mRNA and protein expression as evaluated by RNA sequencing (RNA-seq) and proteomic analysis (Figures S1B–S1D).

To evaluate the translational efficiency of their 5′ UTR, we designed a Luc modRNA construct carrying different 5′ UTRs taken from the selected genes (Figure 2A). We then compared the expression of 5 newly designed, different Luc modRNAs with different 5′ UTRs with our Luc modRNAs that carry artificial 5′ UTRs (36 nt, Luc-Control) and are commonly used for *in vitro* modRNA production (Figure 2B). Our *in vitro* screening showed that all Luc modRNAs carrying different 5′ UTRs allowed protein translation of modRNA in rat neonatal cardiomyocytes (RNCMs). However, in comparison to the Luc-Control, Gsn, Pzp, Serpina, and Fn3k had significantly lower translation, except Luc modRNA carrying 5′ UTR of Ces1d (Luc-Ces1d), which showed a significant increase of 23% in modRNA translation in comparison to Luc-Control (Figure 2C). To evaluate that this result is not due to different transfection efficiency, a parallel *in vitro* experiment was done with cotransfection of firefly Luc modRNA carrying different 5′ UTR modRNAs and renilla Luc modRNA as an internal control that carries control 5′ UTR in neonatal rat CMs. 24 h post-transfection, we used IVIS system to measure simultaneously the translation of the two different luciferase (firefly and renilla) modRNA *in vitro* (Figure S2). Our results show, similar to Figure 2, that Ces1d modRNA gave significantly higher firefly Luc

Figure 1. Characterizing the Ischemic Heart Transcriptome and Proteome

(A) Sham-operated heart or heart, 4 or 24 h post-MI was collected, and the ischemic area tissue (or equivalent area in sham hearts) was divided into two equal pieces. One-half of the ischemic heart was sequenced for transcriptomic analysis (n = 10 total, Sham n = 3, 4 h post-MI n = 3, or 24 h post-MI n = 4), whereas the other one-half of the ischemic heart was evaluated for protein level using mass spectrometry (n = 12 total, Sham n = 4, 4 h post-MI n = 4, or 24 h post-MI n = 4). (B and C) Hierarchical clustering dendrogram of 2,272 genes with corresponding mRNA level (B) or 2,397 protein intensity (C) in Sham, 4 h post-MI or 24 h post-MI hearts. (D and E) Correlation analysis between changes in levels of proteins and mRNA in the LV, 4 (D) or 24 (E) h post-MI. The bottom-right shaded rectangles include genes that show static or reduced mRNA levels post-MI, whereas their encoded protein levels increased with comparison to sham. (F and G) A list of genes that encode for proteins with elevated protein levels (fold change > 2), while showing mRNA downregulated (fold change < 0.64), 4 h (F) or 24 h (G) post-MI. Genes with yellow backgrounds have a 5′ UTR that is shorter than 100 base pairs (see Table S1 for the full sequences of the 5′ UTR of the 5 genes).

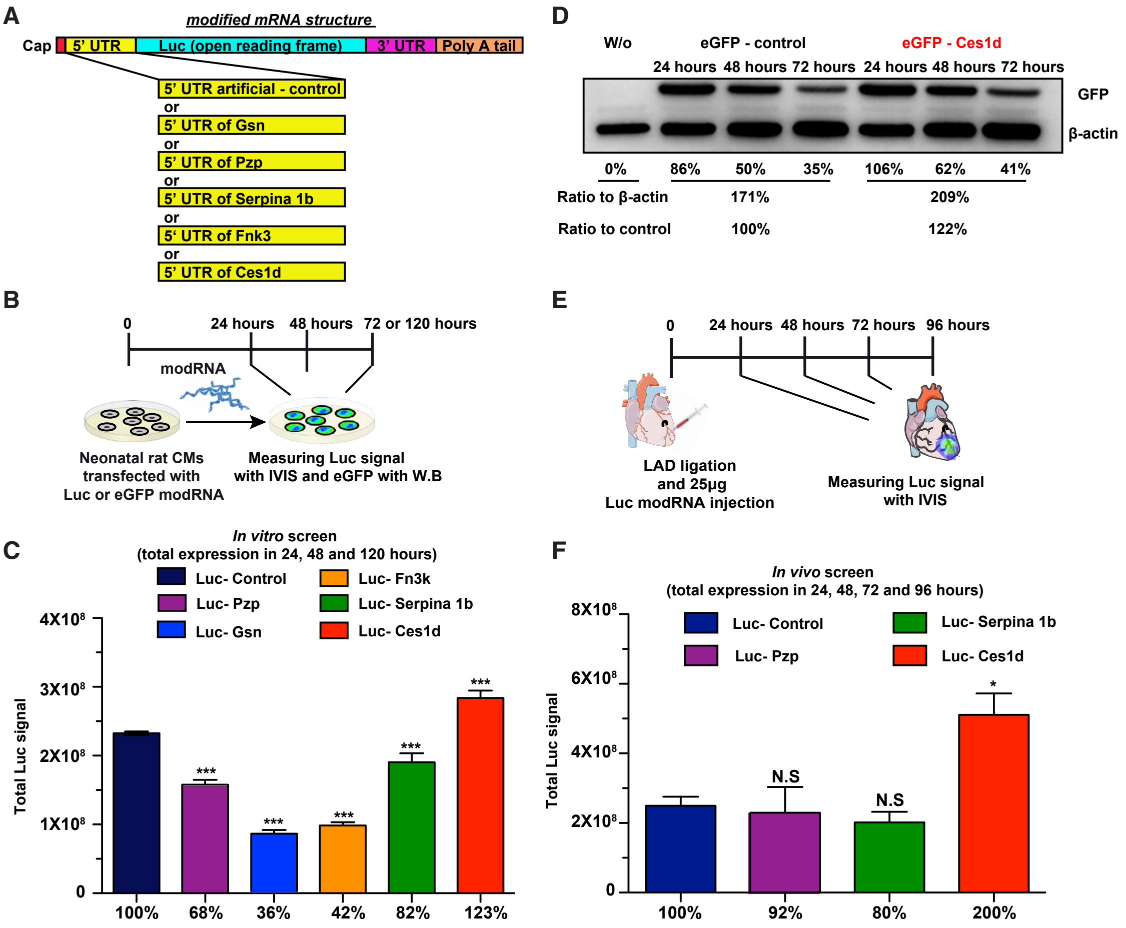

Figure 2. Evaluating the Translation Efficiency of modRNA Carrying Different Potential 5′ UTRs in Neonatal Rat CMs or in a Mouse Heart Ischemic Model
(A) Schematic representation of modRNA structure and the replacement of different potential 5′ UTRs in the Luc modRNA. (B) Experimental plan to evaluate the translation efficiency of Luc or GFP modRNA carrying different potential 5′ UTRs in neonatal rat CMs using IVIS or western blot analysis, respectively. (C and D) Quantification of the IVIS (C; n = 4) and western blot (D) experiments that were described in (B). (E) Experimental plan to evaluate the translation efficiency of Luc modRNA carrying different potential 5′ UTRs in mouse hearts, 24 h post-MI using IVIS analysis. (F) Quantification of the IVIS experiment that was described in (E) (n = 4). One-way ANOVA and Tukey's multiple comparison test were used for (C) and (F). ***p < 0.001, *p < 0.05; N.S, not significant.

modRNA translation with comparison to firefly Luc-Control modRNA, without significant change in renilla Luc modRNA translation. These results indicate that the high translation is due to the use of Ces1d 5′ UTR and not due to different transfection efficiency.

Additionally, to confirm our finding, we made EGFP modRNA, carrying 5′ UTR of Ces1d (EGFP-Ces1d), or EGFP modRNA, carrying artificial 5′ UTR (EGFP-Control) and compared their translation level of EGFP using western blot in neonatal rat CMs. Similar to our previous results, EGFP-Ces1d showed a 22% increase in modRNA translation in comparison to EGFP-Control (Figure 2D).

We decided to evaluate further our Luc modRNA carrying different 5′ UTRs in an MI mouse model. We selected the Luc-5′ UTR modRNAs (Pzp, Serpina 1b, and Ces1d) that showed the highest translation in our *in vitro* assay (Figures 2B–2D), and we measured their translation at 1, 2, and 3 days post-MI (Figure 2E). Our analysis revealed that Luc-Ces1d modRNA had a significantly (2-fold) higher expression in comparison to Luc-Control or Luc modRNA carrying 5′ UTR of Pzp or Serpina (Figure 2F).

To identify if Ces1d 5′ UTR regulates protein translation only in the heart ischemic mouse model, we evaluated both Luc-Ces1d and Luc-Control modRNA translation in a nonischemic heart (Figures 3A and 3B) or ischemic heart mouse models (Figures 3C, 3D, S3A, and S3B). We show that whereas there were no significant changes in the translation level of Luc-Ces1d in comparison with Luc-Control in the heart without MI, 1, 2, and 3 days post-injection, there is a significantly higher translation of Luc-Ces1d, 2 days post-MI in comparison to the Luc-Control. This may indicate that Ces1d enhances modRNA translation in the heart only under an ischemic condition, such as MI.

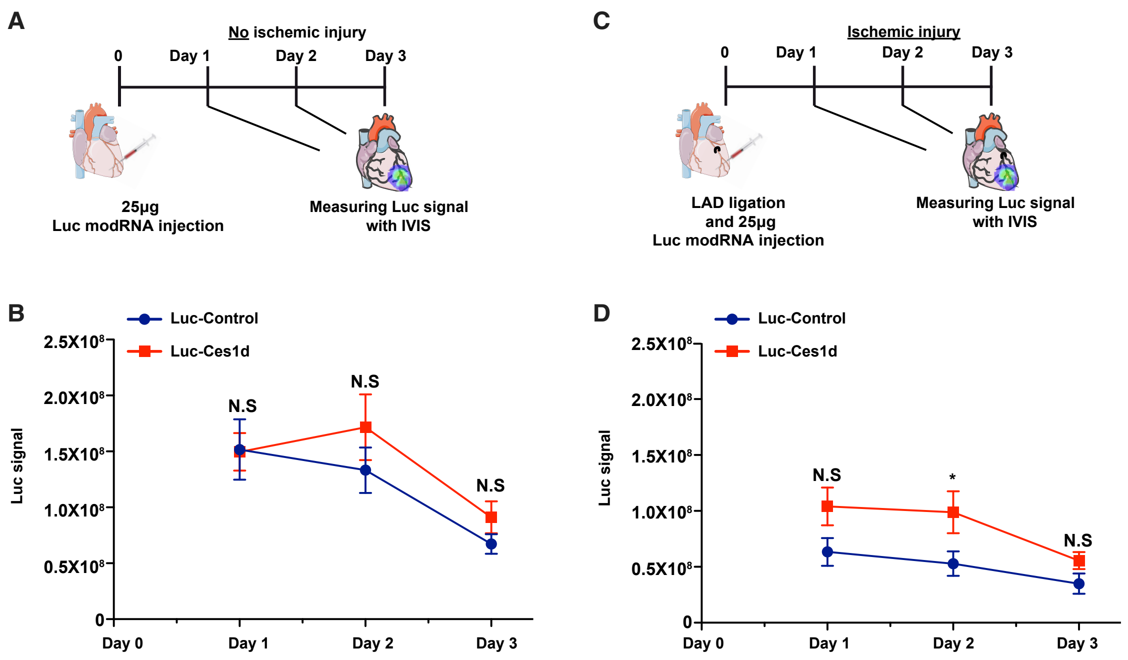

Figure 3. 5′ UTR of Ces1d Enhances Significantly mRNA Translation in Ischemic Heart but Not in a Nonischemic Mouse Model

(A) Experimental plan to evaluate the translation efficiency of Luc modRNA carrying 5′ UTR of Ces1d or artificial (control) 5′ UTR in a nonischemic heart model. (B) Quantification of the experiment that was described in (A) (n = 15). (C) Experimental plan to evaluate the translation efficiency of Luc modRNA carrying 5′ UTR of Ces1d or artificial (control) 5′ UTR in an ischemic heart model. (D) Quantification of the experiment that was described in (C) (n = 15). Two-way ANOVA and Tukey's multiple comparison test were used for (B) and (D). *p < 0.05; N.S, not significant.

We then wanted to evaluate the role of Ces1d in modRNA translation under an ischemic condition in other organs besides the heart. As acute hepatic or renal ischemia may lead to hepatic or renal failure that may be fatal, we decided to choose both liver and kidney as two representative organs for ischemic disease. Similar to the heart, ischemic injury in the liver significantly increases the expression of Luc-Ces1d (4 h post-delivery) in comparison to Luc-Control (Figures 4A–4D, S3C, and S3D). Again, similar to the heart, no significant differences were seen between groups in the liver without ischemic injury (Figures 4A and 4B). On the contrary, Luc-Ces1d had no significant translation differences in comparison to Luc-Control in the ischemic and nonischemic condition in the kidney (Figures 4E–4H, S3E, and S3F).

To identify the RNA element in Ces1d 5′ UTR that is responsible for the significant enhancement of translation of modRNA carrying Ces1d 5′ UTR, we examined the Ces1d 5′ UTR consensus elements that had been conserved among different species (e.g., mouse, rat, pig, gerbil, and human). Interestingly, 4 out of the 5 elements (elements B, C, D, and E) were conserved among species (Figure 5A). Based on this information, we designed a Luc modRNA construct carrying different 5′ UTR elements of Ces1d (elements A–E) and compared its translation ability in neonatal rat CM to Luc-Ces1d (Figures 5B and 5C). Our results indicated a significant reduction in translation ability in elements A, B, C, and E but not in D (Figure 5C). We hypothesized that element D is the RNA element that is responsible for the higher translation ability of Luc-Ces1d. To test our hypothesis, we compared in an ischemic heart model the expression of Luc-Ces1d or Luc-element D with Luc-Control over 3 days (Figure 5D). Our results show that Luc modRNA, carrying 5′ UTR of Ces1d or element D, has higher translation in comparison to Luc-Control (Figure 5E). Intriguingly, element D alone had a significantly higher translation in the heart at day 3 post-MI (Figure 5D). Overall, with the combination of the 3-day readout, Luc-element D has a 2.5-fold higher translation in the heart post-MI in comparison to the widely used artificial 5′ UTR (Luc-Control). However, Luc-element D failed to increase translation over Luc-Ces1d in liver ischemic injury (Figures S4A and S4B) and over Luc-Ces1d or Luc-Control in heart nonischemic injury (Figures S5A and S5B).

DISCUSSION

The use of modRNA as a gene-delivery tool is growing in the field of therapeutic medicine. Our laboratory aims to use modRNA to induce cardiac protection and cardiovascular or cardiac regeneration post-myocardial infarction.[8] Zangi et al.[6] have shown that immediate delivery of vascular endothelial growth factor (VEGF)-A modRNA in a MI mouse model led to induction of cardiovascular regeneration. A follow-up study in large animals shows a significant improvement of cardiac function when VEGF-A modRNA was delivered 1 week post-MI.[40] Recently, it was shown that intradermal delivery of VEGF-A modRNA in patients suffering from type 2 diabetes is safe and may promote angiogenesis.[41] VEGF-A modRNA has now been evaluated in phase 2a human clinical trials to improve cardiac function in

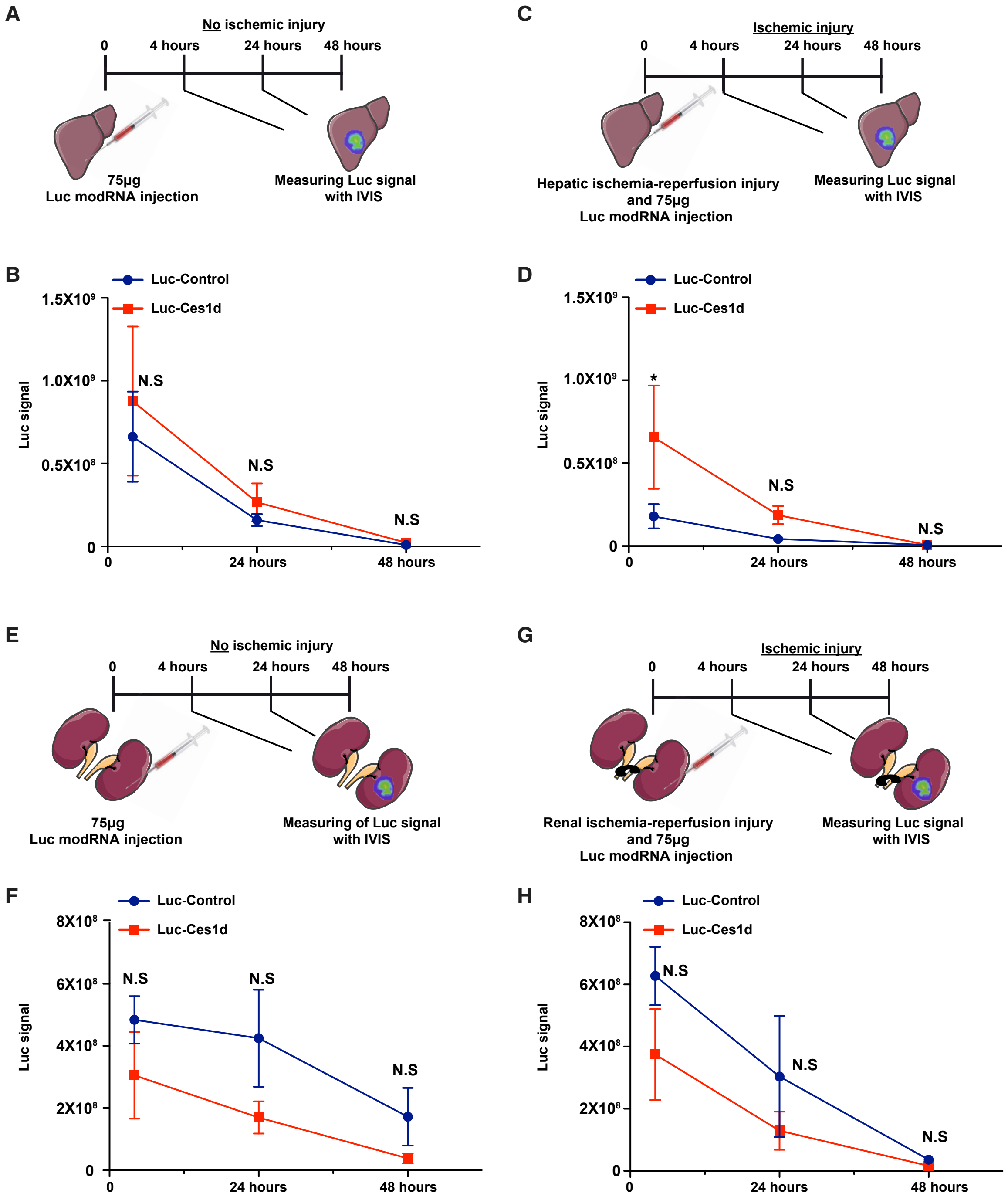

Figure 4. 5′ UTR of Ces1d Enhances Significantly Luc modRNA Translation in Liver but Not in a Nonischemic or Kidney Ischemic Mouse Model
(A) Experimental plan to evaluate the translation efficiency of Luc modRNA carrying 5′ UTR of Ces1d or artificial (control) 5′ UTR in a nonischemic liver model. (B) Quantification of the experiment that was described in (A) (n = 6). (C) Experimental plan to evaluate the translation efficiency of Luc modRNA carrying 5′ UTR of Ces1d or artificial (control) 5′ UTR in an ischemic liver model. (D) Quantification of the experiment that was described in (C) (n = 6). (E) Experimental plan to evaluate the translation efficiency of Luc modRNA carrying 5′ UTR of Ces1d or artificial (control) 5′ UTR in a nonischemic kidney model. (F) Quantification of the experiment that was described in (E) (n = 6). (G) Experimental plan to evaluate the translation efficiency of Luc modRNA carrying 5′ UTR of Ces1d or artificial (control) 5′ UTR in an ischemic kidney model. (H) Quantification of the experiment that was described in (G) (n = 6). Two-way ANOVA and Tukey's multiple comparison test were used for (B), (D), (F), and (H). *$p < 0.05$; N.S, not significant.

A

List of Ces1d 5'UTR RNA elements

Species	Element A	Element B	Element C	Element D	Element E
Mouse	aggaggcgggtcccctggtcca	caacagaagcattgctaaagcag	cagatagc	tcagagacccacagagccc	ttgtccttccaca
Rat	-	tgctaaaggaa (34%)	caaatagc (87.5%)	ccacagatccgcagaaccc (36%)	ttgtccttccaca (100%)
Pig	gaattcacaggatcatatccagtactgtt	caaggacaagtgcatttccatgaatcagga	cagagagc (87.5%)	tccttggctcccaacc	tgttgtcttcccatg
Gerbil	caggaccttgggtcca	caacagcattgctaaagc (77.7%)	agcagata	tcacacagacccacggaaccc (46%)	ttgtccttccaca (100%)
Human	agcgcagggcggtaactctgggcggggctgggctcca	gggctggacagcacagtccctctgaactgca	cagagacctcgc (75%)	aggccccgagaac	tgtcgcccttccacg (100%)
Consensus	gga++++ggggc+cata+ccca+	acagg+c+agaacccat+gcct+ccat+aat	caga+a++aga+	ctccc+ggctcc+a+ca++	ttgtcttcccat

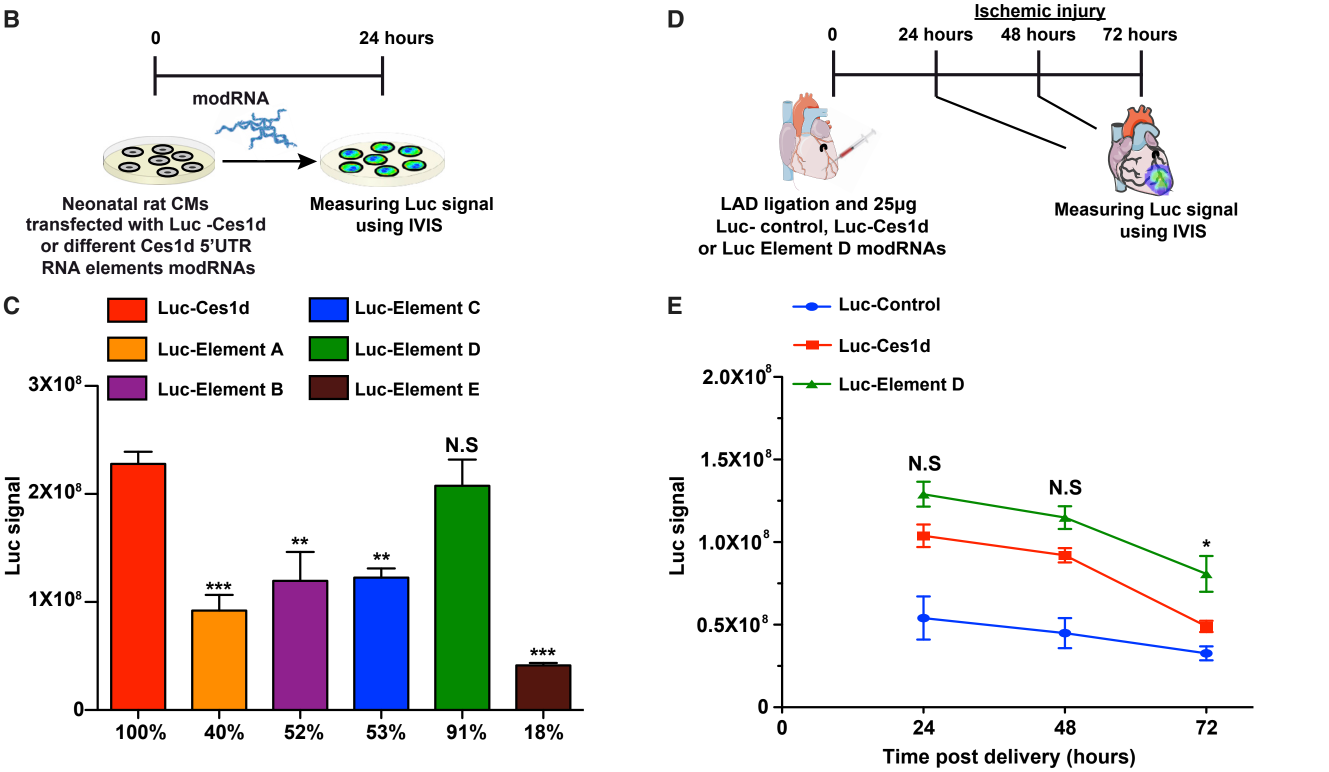

Figure 5. A Specific RNA Element in 5′ UTR of Ces1d Enhances Significantly Luc modRNA Translation in a Heart Ischemic Mouse Model

(A) List of the different RNA elements in 5′ UTR of Ces1d that has been conserved across different species. (B) Experimental plan to evaluate the translation efficiency of different RNA elements in 5′ UTR of Ces1d in neonatal rat CMs using Luc modRNA and IVIS analysis. (C) Quantification of the experiments that were described in (B) (n = 4). (D) Experimental plan to evaluate the translation efficiency of Luc modRNA carrying full-length 5′ UTR of Ces1d, only element D from Ces1d 5′ UTR, or artificial (control) 5′ UTR in an ischemic heart model. (E) Quantification of the experiments that were described in (D) (n = 5). One-way ANOVA and Tukey's multiple comparison test were used for (C). Two-way ANOVA and Tukey's multiple comparison test were used for (E). ***$p < 0.001$, **$p < 0.01$, *$p < 0.05$; N.S, not significant.

heart-failure patients. In parallel, other groups are using the modRNA platform in preclinical studies of different liver diseases to deliver different target genes. The different liver disease models that have been used are for factor IX deficiency hemophilia B,[42] acute intermittent porphyria,[43] glycogen storage disease type 1A,[44] thrombotic thrombocytopenic purpura,[45] alpha-1 antitrypsin deficiency,[46] Crigler-Najjar syndrome type 1,[47] and urea cycle disorder[48] with target genes of FIX, PBGD, glucose-6-phosphatase, ADAMTS13, SERPINA1, bilirubin-UGT, and ornithine transcarbamoylase, respectively. As was described above, both the heart and liver disease have been heavily studied in search for new treatments. One of the obstacles in moving to large animals and clinical trials is the need for a large amount of modRNA to transfect human or large-animal heart and liver. In addition, due to the short expression of modRNA, there might be a need to deliver the modRNA several times. In order to reduce the need for a large amount of modRNA for delivery, we aim to improve modRNA translation so the amount of protein that has been received from the same amount of modRNA will be higher.

Several elements within RNA are responsible for the regulation of translation, including type of nucleotides, poly(A) tail length, or the structure of 5′ UTR, 3′ UTR, and cap analog. We showed that replacement of pseudouridine with N1-Methyl-Pseudouridine-5'-Triphosphate (1-M-pseudouridine) results in a higher modRNA translation in the heart of the latter.[4] In addition we found that a longer poly(A) tail can increase the translation of the modRNA *in vivo* (unpublished data). Here, we describe a unique first-of-this-kind screen, comparing proteomic and transcriptomic analysis to identify the 5′ UTR sequence that can increase the translation of modRNA in an ischemic condition with comparison to the widely used artificial 5′ UTR that has been used in previous modRNA publications.[4–6,14,40,41]

Our data show that there is a positive correlation at both 4 and 24 h post-MI in mRNA levels and protein intensity in the ischemic heart. However, we were able to identify 19 different genes with negative correlation, in which their mRNA level was reduced or unchanged, whereas their protein levels were upregulated, 4 and 24 h post-MI. The average length of 5′ UTRs is ∼100 to ∼220 nt across species.[49] In vertebrates, longer 5′ UTRs tend to be associated with poor translation.[50] Therefore, we decided to select 5 genes with the shortest 5′ UTR (<100 base pairs) (Figure 1).

Negative correlation between mRNA levels and protein expression has been reported, especially upon internal or external stimuli that trigger alteration in translation of specific genes. VEGF has been shown to be a stress-induced protein in many conditions, such as hypoxia and hypoglycemia.[51,52] Other genes in which translation changes in response to stimulation of internal or external signals are platelet-derived growth factor 2 (PDGF2) and transforming growth factor β (TGF-β).[53] During the embryonic stage, when most of the organ development and cell differentiation takes place, translation regulation plays a significant role by altering the levels of expression of specific mRNA subsets during a certain time frame, whereas the bulk of transcripts remain unaffected.[54–56]

In our study, we were able to identify several 5′ UTRs that may allow translation of modRNA in the heart or liver post-ischemic injury. Both Pzp and Serpina 1b 5′ UTR show nonsignificant and similar translation ability as the control 5′ UTR sequence *in vivo* (Figures 2E and 2F). As our control 5′ UTR (artificial 5′ UTR) is the top and most known and commonly used 5′ UTR in the mRNA field,[6,40,57–61] we believe that these results indicate how well, relative to this premium control, our selected 5′ UTR sequences performed.

In this particular work, we focused on the 5′ UTR of Ces1d (Figures 2, 3, and 4) as an enhancer of modRNA translation in cardiac and hepatic ischemic conditions. Ces1d belongs to a family of carboxylesterases that have important roles in lipid metabolism, and they hydrolyze endogenous esters and thioesters. Carboxylesterases are known for involvement in the detoxification of environmental toxicants, as well as the prodrug metabolism. Ces1d is the functional mouse ortholog of human CES1 and has similar protein-expression profiles at different cells or tissues as CES1. Several roles of Ces1d have been reported to be directly associated with lipid metabolism.[62–64] As lipid metabolism is an important process for normal heart function, fatty acids are the preferred substrates under aerobic conditions.[65] As Ces1d association with lipid metabolism and as MI lead to alteration of the lipid metabolism, we hypothesize that Ces1d mRNA has been triggered by the ischemic condition in the heart post-MI, which leads it to translate better. We showed that element D is the RNA element in Ces1d that is responsible for the elevation in mRNA translation post-MI (Figure 4). It will be interesting to evaluate the element D and Ces1d in different ischemic conditions and different organs bedsides the heart, liver, and kidney. The fact that Ces1d gave higher translation in the ischemic heart and liver but not the kidney is interesting, as all three organs primarily use fatty acid oxidation for energy. This may indicate that each organ and physiological condition will need a separate evaluation using a similar approach as ours.

In conclusion, we have identified the 5′ UTR of Ces1d and RNA element D in Ces1d 5′ UTR as RNA elements for improving modRNA translation in the heart and liver post-ischemic injury. This may have clinical applications, as both organs have been heavily targeted with modRNA in different cardiac and hepatic diseases. Our results may contribute to designing superior modRNA for preclinical studies in ischemic cardiac and liver diseases, carrying 5′ UTR of Ces1d or RNA element D.

METHODS

Mice

All animal procedures were performed according to protocols approved by the Icahn School of Medicine at Mount Sinai Institutional Care and Use Committee. Swiss Webster (CFW) mice were used for the study. Before surgery, mice were anesthetized with a cocktail of 100 mg/kg ketamine and 10 mg/kg xylazine. For protein expression, mice were injected with 25 μg of modRNA in citrate buffer directly into the myocardium during open-chest surgery. When required, 25 μg of modRNA was injected into the border zone with three injections immediately after left anterior descending artery (LAD) ligation.

modRNA Synthesis

Clean PCR products generated with plasmid templates (GeneArt; Thermo Fisher Scientific) were used as the template for mRNA. modRNAs were generated by transcription *in vitro* with a customized ribonucleoside blend of anti-reverse cap analog, 3′-O-Me-m^{7}G(5′)ppp(5′)G (6 mM; TriLink Biotechnologies), guanosine triphosphate (1.5 mM; Life Technologies), adenosine triphosphate (7.5 mM; Life Technologies), cytidine triphosphate (7.5 mM; Life Technologies), and N1-Methyl-Pseudouridine-5'-Triphosphate (7.5 mM; TriLink Biotechnologies). The mRNA was purified with the MEGAclear kit (Life Technologies) and treated with Antarctic Phosphatase (New England Biolabs). It was then repurified with the MEGAclear kit. The mRNA was quantified on a NanoDrop spectrometer (Thermo Scientific), precipitated with ethanol and ammonium acetate, and resuspended in 10 mM Tris-HCl and 1 mM EDTA.

Rat Neonatal CM Isolation

Ventricular RNCMs were isolated from 3- to 4-day-old Sprague-Dawley rat pups by a Pierce primary cardiomyocytes isolation kit (Thermo Fisher Scientific; catalog number 88282). After isolation, cells were incubated in 10% horse serum DMEM and change media, and a cardiomyocyte growth supplement was added after 16 h and transfected with modRNA.

In Vitro modRNA Transfection

2.5 μg/well of a 24-well plate of Luc modRNA or 10 μg/well of a 6-well plate of nuclear GFP (nGFP) modRNA was transfected into neonatal rat CMs using the transfection reagent jetPEI (Polyplus).The transfection mixture was prepared according to the manufacturer's protocol, and then it was added to cells cultured in DMEM containing 10% fetal bovine serum (FBS) and antibiotic-antimycotic (anti-anti). Then, 24 h post-transfection, the expression level of cells was imaged and measured in IVIS, or cell lysates were collected and analyzed by western blot.

Heart Ischemic Injury

MI was induced by permanent ligation of the LAD. The left thoracic region was shaved and sterilized. After intubation, the heart was exposed by left thoracotomy. The LAD was ligated with a suture. Mouse hearts without (sham) or with ischemic injury (MI) were collected at 4 and 24 h post-MI (Figure 1A). The ischemic area tissue (or equivalent area in sham hearts) was collected and divided into two pieces and snap frozen quickly. One-half of the ischemic heart tissues was sent for RNA-seq, whereas the other one-half was sent for proteomics analysis to evaluate gene and protein fold change, respectively, between MI and sham hearts. When required, we injected 25 μg modRNA into the infarct border zone immediately after LAD ligation. The thoracotomy and skin were sutured closed in layers. Excess air was removed from the thoracic cavity, and the mouse was removed from ventilation when normal breathing was established.

Liver Ischemic Injury

Liver ischemia was induced by closing the left lateral lobe and median for 1 h, and 25 μg modRNA was injected into the left lateral lobe in three different injections immediately after clip was removed.

Kidney Ischemic Injury

Kidney ischemia was induced by applying a micro clip onto the renal artery and renal vein. Successful ischemia can be visually confirmed by a gradual uniform darkening of the kidney. The clip was removed after 30 min, and 25 μg of modRNA was injected in the kidney with three injections.

In Vivo modRNA Delivery

Luc modRNA (25 μg) in a total volume of 60 μL in Tris-borate (TB) buffer was delivered via direct injection to the myocardium. The sucrose-citrate buffer contains 20 μL sucrose in nuclease-free water (0.3 g/mL) and 20 μL citrate (0.1 M, pH 7; Sigma) mixed with 20 μL modRNA. The transfection mixture was directly injected (three individual injections, 20 μL each) into the heart, kidney, or liver.

Detection of Luciferase Expression Using the IVIS System

Bioluminescence imaging of the transfected cells (24–72 h) or injected mice was taken at different time points (4–144 h) in the IVIS system. To visualize cells expressing firefly luciferase *in vitro*, D-luciferin was added to the cell-culture plate, and an image was taken in the IVIS system (IVIS Spectrum National Center for Research Resources [NCRR] S10-RR026561-01 at the Preclinical Small Imaging Core at Mount Sinai Medical Center). To visualize cells expressing renilla luciferase *in vitro*, cells were washed twice with media, renilla luciferase substrate was added to the cell-culture plate, and an image was taken in emission filter 500. To visualize tissues expressing Luc *in vivo*, mice were anesthetized with isoflurane (Abbott Laboratories), and luciferin (150 μg/g body weight; Sigma) was injected intraperitoneally. Mice were imaged using an IVIS imaging system (IVIS Spectrum NCRR S10-RR026561-01 at the Preclinical Small Imaging Core at Mount Sinai Medical Center) every 2 min until the Luc signal reached a plateau. Imaging data were analyzed and quantified with Living Image software.

Western Blotting

Cell lysates were collected and subjected to SDS-PAGE in 12% precast NuPAGE Bis/Tris gels (Invitrogen) under reducing conditions in MES running buffer (Invitrogen). The resulting bands were transferred onto a nitrocellulose membrane (Bio-Rad) by blotting in a semidry transfer apparatus with NuPAGE-MOPS (3-(*N*-morpholino)propanesulfonic acid) transfer buffer (Invitrogen). The membrane was blocked by incubation with Tris-buffered saline (TBS)/Tween containing 5% dry milk powder and incubated with specific primary antibodies overnight at 4°C. It was then washed in TBS/Tween and incubated with rabbit or goat secondary antibodies conjugated to horseradish peroxidase for 1 h at room temperature. Antibody binding was detected with an enhanced chemiluminescence (ECL) detection system (Pierce). We used prestained protein standards (Amersham) to determine molecular weight.

RNA Isolation

Total RNA was isolated with an RNeasy Mini Kit (QIAGEN), and DNA was degraded by treatment with TURBO DNase (Invitrogen). RNA quality was checked by a bioanalyzer.

RNA Sequencing

Poly(A)-tailed RNA was prepared by the Epigenomics Core at Cornell Medical College, with the mRNA Seq Sample Prep Kit (Illumina) and used to create libraries for HiSeq 2000 sequencing (Illumina). We used single, 50-bp reads for sequencing. We obtained a mean of 30 million reads per sample, with a mean quality score of 35.2. We used Partek flow software for data analysis. RNA-seq reads were aligned to mm10 with STAR version 2.53a. Read counts were generated by the application of the Partek expectation-maximization (E/M) algorithm to University of California, Santa Cruz (UCSC), RefSeq 2017-08-02. Counts were normalized with trimmed mean of maximum (TMM)-values algorithm, and the Partek flow gravitational search algorithm (GSA) was used for statistical analysis. The RNA-seq data used in this study is available using GenBank: GSE138201.

Protein Mass Spectrometry

All solvents were high-performance liquid chromatography (HPLC) grade from Sigma-Aldrich, and all chemicals, where not stated otherwise, were obtained from Sigma-Aldrich. For ample preparation, samples were lysed in Biognosys' lysis buffer with a TissueLyser II bead mill (QIAGEN) using stainless-steel grinding beads for 3 min at 30 Hz. Samples were treated with benzonase after lysis to reduce DNA contamination. Protein concentrations of the lysates were estimated using a UV-VIS (visible) spectrometer (SPECTROstar Nano, BMG Labtech). Approximately 100 μg of protein from each sample was denatured using Biognosys' denature buffer, reduced using Biognosys' reduction solution for 60 min at 37°C and alkylated using Biognosys' alkylation solution for 30 min at room temperature in the dark. Subsequently, digestion to peptides was carried out using 3 μg of trypsin (Promega) overnight at 37°C.

Peptides were desalted using C18 MacroSpin columns (The Nest Group), according to the manufacturer's instructions, and dried down using a SpeedVac system. Peptides were resuspended in 50 μL liquid chromatography (LC) solvent A (1% acetonitrile, 0.1% formic acid [FA]) and spiked with Biognosys' iRT (indexed retention time) kit calibration peptides. Peptide concentrations were determined using a UV-VIS spectrometer (SPECTROstar Nano, BMG Labtech).

For high pH reverse-phase (HPRP) fractionation, equal volumes of samples from each condition were pooled. Ammonium hydroxide was added to all pools to a pH value > 10. The fractionation was performed using a Dionex UltiMate 3000 RS Pump (Thermo Scientific) on an Acquity UPLC CSH (ultra-performance liquid chromatography charged surface hybrid) C18 1.7 μm, 2.1 × 150 mm column (Waters). The gradient was 2% to 35% solvent B in 10 min; solvents were the following: A, 20 mM ammonium formate in H_2O, and B, acetonitrile. Fractions were taken every 15 s and sequentially pooled to 4 fraction pools. These were dried down and resolved in 20 μL solvent A. Prior to mass spectrometric analyses, they were spiked with Biognosys' iRT kit calibration peptides. Peptide concentrations were determined using a UV-VIS spectrometer (SPECTROstar Nano, BMG Labtech).

For the liquid chromatography-tandem mass spectrometry (LC-MS/MS; shotgun) measurements, 2 μg of peptides was injected to an in-house packed C18 column (ReproSil-Pur, Dr. Maisch GmbH; 1.9 μm particle size, 120 Å pore size, 75 μm inner diameter, 50 cm length; New Objective) on a Thermo Scientific EASY-nLC 1200 nano-liquid chromatography system connected to a Thermo Scientific Q Exactive HF mass spectrometer equipped with a standard nano-electrospray source. LC solvents were the following: A, 1% acetonitrile in water with 0.1% FA, and B, 15% water in acetonitrile with 0.1% FA. The nonlinear LC gradient was 1%–55% solvent B in 60 min, followed by 55%–90% B in 10 s, 90% B for 10 min, 90%−100% B in 10 s, and 1% B for 5 min. A modified top 15 method was used.[66]

The mass spectrometric data were analyzed using Biognosys' search engine Pulsar (version 1.0.16105), and the false-discovery rate on peptide and protein level was set to 1%. A mouse UniProt FASTA database (*Mus musculus*, 2017-07-01) was used for the search engine, allowing for 2 missed cleavages and variable modifications (N-terminal acetylation, methionine oxidation).

For the LC-MS/MS heart-rate monitor (HRM) measurements, 2 μg of peptides per sample was injected to the C18 column (ReproSil-Pur, Dr. Maisch GmbH; 1.9 μm particle size, 120 Å pore size, 75 μm inner diameter, 50 cm length; New Objective) on a Thermo Scientific EASY-nLC 1200 nano-liquid chromatography system connected to a Thermo Scientific Q Exactive HF mass spectrometer equipped with a standard nano-electrospray source. LC solvents were the following: A, 1% acetonitrile in water with 0.1% formic acid, and B, 15% water in acetonitrile with 0.1% formic acid. The nonlinear LC gradient was 1%–52% solvent B in 60 min, followed by 52%−90% B in 10 s, and 90% B for 10 min. A data-independent acquisition (DIA) method with one full-range survey scan and 14 DIA windows was used.

HRM mass spectrometric data were analyzed using Spectronaut Pulsar software (Biognosys). The false-discovery rate on peptide and protein level was set to 1%, and data were filtered using row-based extraction. The assay library (protein inventory) generated in this project was used for the analysis. The HRM measurements analyzed with Spectronaut were normalized using local regression normalization.[67]

Merging Proteomics and Transcriptomics Data

For proteomics/transcriptomics merging, gene IDs contained in the transcriptomics datasets were matched to gene names in the mouse UniProt Swiss-Prot proteome.

Statistical Analysis for Luciferase Activity Assay and RNA Protein Correlation

All statistical analysis was performed with GraphPad Prism software. Values are reported as mean ± SD. One-way ANOVA with Bonferroni correction (*$p < 0.05$ considered significant) was used for comparisons among groups. Pearson's R correlation was calculated for correlation between changes in mRNA expression and protein levels.

SUPPLEMENTAL INFORMATION

Supplemental Information can be found online at https://doi.org/10.1016/j.omtm.2020.03.019.

AUTHOR CONTRIBUTIONS

N.S. designed the study, carried out most of the experiments, analyzed most of the data, and wrote the manuscript. Y.H. designed, carried out, and analyzed the RNA-seq and proteomics experiments and revised the manuscript. M.T.K.S. prepared modRNAs and performed experiments. K.K. performed experiments and revised the manuscript. A.M. revised the manuscript. N.H., B.A., and S.A. performed experiments and helped analyze the data. E.C. carried out all surgery and modRNA injections in mouse models. L.Z. designed the experiments, analyzed data, and wrote the manuscript.

CONFLICTS OF INTEREST

L.Z., N.S., and Y.H. are inventors of a provisional patent, which covers the results in this manuscript.

ACKNOWLEDGMENTS

We thank the Preclinical Small Imaging Core at Mount Sinai Medical Center, especially Zhou Yu for technical assistance in bioluminescence imaging. We also thank Shaira Ahmed, Ragy Beshay, and Asharee Mahmood for their assistance in the experiment. This work was funded by a cardiology start-up grant awarded to the Zangi laboratory and also by NIH grant R01 HL142768-01.

REFERENCES

1. Dargie, H. (2005). Heart failure post-myocardial infarction: a review of the issues. Heart *91*, ii3–ii6.
2. Go, A.S., Mozaffarian, D., Roger, V.L., Benjamin, E.J., Berry, J.D., Blaha, M.J., Dai, S., Ford, E.S., Fox, C.S., Franco, S., et al.; American Heart Association Statistics Committee and Stroke Statistics Subcommittee (2014). Heart disease and stroke statistics–2014 update: a report from the American Heart Association. Circulation *129*, e28–e292.
3. Tilemann, L., Ishikawa, K., Weber, T., and Hajjar, R.J. (2012). Gene therapy for heart failure. Circ. Res. *110*, 777–793.
4. Sultana, N., Magadum, A., Hadas, Y., Kondrat, J., Singh, N., Youssef, E., Calderon, D., Chepurko, E., Dubois, N., Hajjar, R.J., and Zangi, L. (2017). Optimizing Cardiac Delivery of Modified mRNA. Mol. Ther. *25*, 1306–1315.
5. Hadas, Y., Sultana, N., Youssef, E., Sharkar, M.T.K., Kaur, K., Chepurko, E., and Zangi, L. (2019). Optimizing modified mRNA in vitro synthesis protocol for heart gene therapy. Mol. Ther. Methods Clin. Dev. *14*, 300–305.
6. Zangi, L., Lui, K.O., von Gise, A., Ma, Q., Ebina, W., Ptaszek, L.M., Später, D., Xu, H., Tabebordbar, M., Gorbatov, R., et al. (2013). Modified mRNA directs the fate of heart progenitor cells and induces vascular regeneration after myocardial infarction. Nat. Biotechnol. *31*, 898–907.
7. Magadum, A., Kaur, K., and Zangi, L. (2019). mRNA-Based Protein Replacement Therapy for the Heart. Mol. Ther. *27*, 785–793.
8. Hadas, Y., Katz, M.G., Bridges, C.R., and Zangi, L. (2017). Modified mRNA as a therapeutic tool to induce cardiac regeneration in ischemic heart disease. Wiley Interdiscip. Rev. Syst. Biol. Med. *9*, 1–18.
9. Weissman, D., and Karikó, K. (2015). mRNA: Fulfilling the Promise of Gene Therapy. Mol. Ther. *23*, 1416–1417.
10. Bobbin, M.L., and Rossi, J.J. (2016). RNA Interference (RNAi)-Based Therapeutics: Delivering on the Promise? Annu. Rev. Pharmacol. Toxicol. *56*, 103–122.
11. Stein, C.A., and Castanotto, D. (2017). FDA-Approved Oligonucleotide Therapies in 2017. Mol. Ther. *25*, 1069–1075.
12. Pardi, N., Tuyishime, S., Muramatsu, H., Kariko, K., Mui, B.L., Tam, Y.K., Madden, T.D., Hope, M.J., and Weissman, D. (2015). Expression kinetics of nucleoside-modified mRNA delivered in lipid nanoparticles to mice by various routes. J. Control. Release *217*, 345–351.
13. Mahiny, A.J., Dewerth, A., Mays, L.E., Alkhaled, M., Mothes, B., Malaeksefat, E., Loretz, B., Rottenberger, J., Brosch, D.M., Reautschnig, P., et al. (2015). In vivo genome editing using nuclease-encoding mRNA corrects SP-B deficiency. Nat. Biotechnol. *33*, 584–586.
14. Kormann, M.S., Hasenpusch, G., Aneja, M.K., Nica, G., Flemmer, A.W., Herber-Jonat, S., Huppmann, M., Mays, L.E., Illenyi, M., Schams, A., et al. (2011). Expression of therapeutic proteins after delivery of chemically modified mRNA in mice. Nat. Biotechnol. *29*, 154–157.
15. Zimmermann, O., Homann, J.M., Bangert, A., Müller, A.M., Hristov, G., Goeser, S., Wiehe, J.M., Zittrich, S., Rottbauer, W., Torzewski, J., et al. (2012). Successful use of mRNA-nucleofection for overexpression of interleukin-10 in murine monocytes/macrophages for anti-inflammatory therapy in a murine model of autoimmune myocarditis. J. Am. Heart Assoc. *1*, e003293.
16. Mignone, F., Gissi, C., Liuni, S., and Pesole, G. (2002). Untranslated regions of mRNAs. Genome Biol. *3*, reviews0004.1–0004.10.
17. Vogel, C., Abreu, Rde.S., Ko, D., Le, S.Y., Shapiro, B.A., Burns, S.C., Sandhu, D., Boutz, D.R., Marcotte, E.M., and Penalva, L.O. (2010). Sequence signatures and mRNA concentration can explain two-thirds of protein abundance variation in a human cell line. Mol. Syst. Biol. *6*, 400.
18. Ong, C.K., Leong, C., Tan, P.H., Van, T., and Huynh, H. (2007). The role of 5′ untranslated region in translational suppression of OKL38 mRNA in hepatocellular carcinoma. Oncogene *26*, 1155–1165.
19. Leppek, K., Das, R., and Barna, M. (2018). Functional 5′ UTR mRNA structures in eukaryotic translation regulation and how to find them. Nat. Rev. Mol. Cell Biol. *19*, 158–174.
20. van der Velden, A.W., and Thomas, A.A. (1999). The role of the 5′ untranslated region of an mRNA in translation regulation during development. Int. J. Biochem. Cell Biol. *31*, 87–106.
21. van Oers, M.M., Vlak, J.M., Voorma, H.O., and Thomas, A.A.M. (1999). Role of the 3′ untranslated region of baculovirus p10 mRNA in high-level expression of foreign genes. J. Gen. Virol. *80*, 2253–2262.
22. Thekkumkara, T.J., Thomas, W.G., Motel, T.J., and Baker, K.M. (1998). Functional role for the angiotensin II receptor (AT1A) 3′-untranslated region in determining cellular responses to agonist: evidence for recognition by RNA binding proteins. Biochem. J. *329*, 255–264.
23. Chen, J.H., Zhang, R.H., Lin, S.L., Li, P.F., Lan, J.J., Song, S.S., Gao, J.M., Wang, Y., Xie, Z.J., Li, F.C., and Jiang, S.J. (2018). The Functional Role of the 3′ Untranslated Region and Poly(A) Tail of Duck Hepatitis A Virus Type 1 in Viral Replication and Regulation of IRES-Mediated Translation. Front. Microbiol. *9*, 2250.
24. Chartier, A., Klein, P., Pierson, S., Barbezier, N., Gidaro, T., Casas, F., Carberry, S., Dowling, P., Maynadier, L., Bellec, M., et al. (2015). Mitochondrial dysfunction reveals the role of mRNA poly(A) tail regulation in oculopharyngeal muscular dystrophy pathogenesis. PLoS Genet. *11*, e1005092.
25. Crawford, E.K., Ensor, J.E., Kalvakolanu, I., and Hasday, J.D. (1997). The role of 3′ poly(A) tail metabolism in tumor necrosis factor-alpha regulation. J. Biol. Chem. *272*, 21120–21127.
26. Nie, L., Khan, I., Misquitta, C.M., and Grover, A.K. (2004). Sarcoplasmic reticulum Ca2+ pump mRNA stability in cardiac and smooth muscle: role of poly A+ tail length. Cell Calcium *35*, 479–484.
27. Peng, Y.H., Lin, C.H., Lin, C.N., Lo, C.Y., Tsai, T.L., and Wu, H.Y. (2016). Characterization of the Role of Hexamer AGUAAA and Poly(A) Tail in Coronavirus Polyadenylation. PLoS ONE *11*, e0165077.
28. Galloway, A., and Cowling, V.H. (2019). mRNA cap regulation in mammalian cell function and fate. Biochim. Biophys. Acta. Gene Regul. Mech. *1862*, 270–279.
29. Grudzien-Nogalska, E., Stepinski, J., Jemielity, J., Zuberek, J., Stolarski, R., Rhoads, R.E., and Darzynkiewicz, E. (2007). Synthesis of anti-reverse cap analogs (ARCAs) and their applications in mRNA translation and stability. Methods Enzymol. *431*, 203–227.
30. Meaux, S., and Van Hoof, A. (2006). Yeast transcripts cleaved by an internal ribozyme provide new insight into the role of the cap and poly(A) tail in translation and mRNA decay. RNA *12*, 1323–1337.
31. Mukherjee, C., Patil, D.P., Kennedy, B.A., Bakthavachalu, B., Bundschuh, R., and Schoenberg, D.R. (2012). Identification of cytoplasmic capping targets reveals a role for cap homeostasis in translation and mRNA stability. Cell Rep. *2*, 674–684.
32. Pfeiffer, B.D., Truman, J.W., and Rubin, G.M. (2012). Using translational enhancers to increase transgene expression in Drosophila. Proc. Natl. Acad. Sci. USA *109*, 6626–6631.
33. Wilkie, G.S., Dickson, K.S., and Gray, N.K. (2003). Regulation of mRNA translation by 5′- and 3′-UTR-binding factors. Trends Biochem. Sci. *28*, 182–188.
34. Chatterjee, S., and Pal, J.K. (2009). Role of 5′- and 3′-untranslated regions of mRNAs in human diseases. Biol. Cell *101*, 251–262.
35. Hinnebusch, A.G., Ivanov, I.P., and Sonenberg, N. (2016). Translational control by 5′-untranslated regions of eukaryotic mRNAs. Science *352*, 1413–1416.

36. Araujo, P.R., Yoon, K., Ko, D., Smith, A.D., Qiao, M., Suresh, U., Burns, S.C., and Penalva, L.O. (2012). Before It Gets Started: Regulating Translation at the 5′ UTR. Comp. Funct. Genomics *2012*, 475731.

37. Dvir, S., Velten, L., Sharon, E., Zeevi, D., Carey, L.B., Weinberger, A., and Segal, E. (2013). Deciphering the rules by which 5′-UTR sequences affect protein expression in yeast. Proc. Natl. Acad. Sci. USA *110*, E2792–E2801.

38. Warren, L., Manos, P.D., Ahfeldt, T., Loh, Y.H., Li, H., Lau, F., Ebina, W., Mandal, P.K., Smith, Z.D., Meissner, A., et al. (2010). Highly efficient reprogramming to pluripotency and directed differentiation of human cells with synthetic modified mRNA. Cell Stem Cell *7*, 618–630.

39. Asrani, K.H., Farelli, J.D., Stahley, M.R., Miller, R.L., Cheng, C.J., Subramanian, R.R., and Brown, J.M. (2018). Optimization of mRNA untranslated regions for improved expression of therapeutic mRNA. RNA Biol. *15*, 756–762.

40. Carlsson, L., Clarke, J.C., Yen, C., Gregoire, F., Albery, T., Billger, M., Egnell, A.C., Gan, L.M., Jennbacken, K., Johansson, E., et al. (2018). Biocompatible, Purified *VEGF-A* mRNA Improves Cardiac Function after Intracardiac Injection 1 Week Post-myocardial Infarction in Swine. Mol. Ther. Methods Clin. Dev. *9*, 330–346.

41. Gan, L.M., Lagerström-Fermér, M., Carlsson, L.G., Arfvidsson, C., Egnell, A.C., Rudvik, A., Kjaer, M., Collén, A., Thompson, J.D., Joyal, J., et al. (2019). Intradermal delivery of modified mRNA encoding VEGF-A in patients with type 2 diabetes. Nat. Commun. *10*, 871.

42. DeRosa, F., Guild, B., Karve, S., Smith, L., Love, K., Dorkin, J.R., Kauffman, K.J., Zhang, J., Yahalom, B., Anderson, D.G., and Heartlein, M.W. (2016). Therapeutic efficacy in a hemophilia B model using a biosynthetic mRNA liver depot system. Gene Ther. *23*, 699–707.

43. Jiang, L., Berraondo, P., Jericó, D., Guey, L.T., Sampedro, A., Frassetto, A., Benenato, K.E., Burke, K., Santamaría, E., Alegre, M., et al. (2018). Systemic messenger RNA as an etiological treatment for acute intermittent porphyria. Nat. Med. *24*, 1899–1909.

44. Roseman, D.S., Khan, T., Rajas, F., Jun, L.S., Asrani, K.H., Isaacs, C., Farelli, J.D., and Subramanian, R.R. (2018). G6PC mRNA Therapy Positively Regulates Fasting Blood Glucose and Decreases Liver Abnormalities in a Mouse Model of Glycogen Storage Disease 1a. Mol. Ther. *26*, 814–821.

45. Liu-Chen, S., Connolly, B., Cheng, L., Subramanian, R.R., and Han, Z. (2018). mRNA treatment produces sustained expression of enzymatically active human ADAMTS13 in mice. Sci. Rep. *8*, 7859.

46. Connolly, B., Isaacs, C., Cheng, L., Asrani, K.H., and Subramanian, R.R. (2018). SERPINA1 mRNA as a Treatment for Alpha-1 Antitrypsin Deficiency. J. Nucleic Acids *2018*, 8247935.

47. Apgar, J.F., Tang, J.P., Singh, P., Balasubramanian, N., Burke, J., Hodges, M.R., Lasaro, M.A., Lin, L., Miliard, B.L., Moore, K., et al. (2018). Quantitative Systems Pharmacology Model of hUGT1A1-modRNA Encoding for the UGT1A1 Enzyme to Treat Crigler-Najjar Syndrome Type 1. CPT Pharmacometrics Syst. Pharmacol. *7*, 404–412.

48. Prieve, M.G., Harvie, P., Monahan, S.D., Roy, D., Li, A.G., Blevins, T.L., Paschal, A.E., Waldheim, M., Bell, E.C., Galperin, A., et al. (2018). Targeted mRNA Therapy for Ornithine Transcarbamylase Deficiency. Mol. Ther. *26*, 801–813.

49. Pesole, G., Mignone, F., Gissi, C., Grillo, G., Licciulli, F., and Liuni, S. (2001). Structural and functional features of eukaryotic mRNA untranslated regions. Gene *276*, 73–81.

50. Davuluri, R.V., Suzuki, Y., Sugano, S., and Zhang, M.Q. (2000). CART classification of human 5′ UTR sequences. Genome Res. *10*, 1807–1816.

51. Shweiki, D., Neeman, M., Itin, A., and Keshet, E. (1995). Induction of vascular endothelial growth factor expression by hypoxia and by glucose deficiency in multicell spheroids: implications for tumor angiogenesis. Proc. Natl. Acad. Sci. USA *92*, 768–772.

52. Akiri, G., Nahari, D., Finkelstein, Y., Le, S.Y., Elroy-Stein, O., and Levi, B.Z. (1998). Regulation of vascular endothelial growth factor (VEGF) expression is mediated by internal initiation of translation and alternative initiation of transcription. Oncogene *17*, 227–236.

53. Tobin, S.W., Douville, K., Benbow, U., Brinckerhoff, C.E., Memoli, V.A., and Arrick, B.A. (2002). Consequences of altered TGF-beta expression and responsiveness in breast cancer: evidence for autocrine and paracrine effects. Oncogene *21*, 108–118.

54. Jackson, R.J., Hellen, C.U., and Pestova, T.V. (2010). The mechanism of eukaryotic translation initiation and principles of its regulation. Nat. Rev. Mol. Cell Biol. *11*, 113–127.

55. Sonenberg, N., and Hinnebusch, A.G. (2009). Regulation of translation initiation in eukaryotes: mechanisms and biological targets. Cell *136*, 731–745.

56. Gebauer, F., and Hentze, M.W. (2004). Molecular mechanisms of translational control. Nat. Rev. Mol. Cell Biol. *5*, 827–835.

57. Kondrat, J., Sultana, N., and Zangi, L. (2017). Synthesis of Modified mRNA for Myocardial Delivery. Methods Mol. Biol. *1521*, 127–138.

58. Lui, K.O., Zangi, L., Silva, E.A., Bu, L., Sahara, M., Li, R.A., Mooney, D.J., and Chien, K.R. (2013). Driving vascular endothelial cell fate of human multipotent Isl1+ heart progenitors with VEGF modified mRNA. Cell Res. *23*, 1172–1186.

59. Magadum, A., Singh, N., Kurian, A.A., Sharkar, M.T.K., Chepurko, E., and Zangi, L. (2018). Ablation of a Single N-Glycosylation Site in Human FSTL 1 Induces Cardiomyocyte Proliferation and Cardiac Regeneration. Mol. Ther. Nucleic Acids *13*, 133–143.

60. Mohamed, T.M.A., Ang, Y.S., Radzinsky, E., Zhou, P., Huang, Y., Elfenbein, A., Foley, A., Magnitsky, S., and Srivstava, D. (2018). Regulation of Cell Cycle to Stimulate Adult Cardiomyocyte Proliferation and Cardiac Regeneration. Cell *173*, 104–116.e12.

61. Turnbull, I.C., Eltoukhy, A.A., Fish, K.M., Nonnenmacher, M., Ishikawa, K., Chen, J., Hajjar, R.J., Anderson, D.G., and Costa, K.D. (2016). Myocardial Delivery of Lipidoid Nanoparticle Carrying modRNA Induces Rapid and Transient Expression. Mol. Ther. *24*, 66–75.

62. Dominguez, E., Galmozzi, A., Chang, J.W., Hsu, K.L., Pawlak, J., Li, W., Godio, C., Thomas, J., Partida, D., Niessen, S., et al. (2014). Integrated phenotypic and activity-based profiling links Ces3 to obesity and diabetes. Nat. Chem. Biol. *10*, 113–121.

63. Lian, J., Watts, R., Quiroga, A.D., Beggs, M.R., Alexander, R.T., and Lehner, R. (2019). Ces1d deficiency protects against high-sucrose diet-induced hepatic triacylglycerol accumulation. J. Lipid Res. *60*, 880–891.

64. Marrades, M.P., González-Muniesa, P., Martínez, J.A., and Moreno-Aliaga, M.J. (2010). A dysregulation in CES1, APOE and other lipid metabolism-related genes is associated to cardiovascular risk factors linked to obesity. Obes. Facts *3*, 312–318.

65. Ford, D.A. (2002). Alterations in myocardial lipid metabolism during myocardial ischemia and reperfusion. Prog. Lipid Res. *41*, 6–26.

66. Scheltema, R.A., Hauschild, J.P., Lange, O., Hornburg, D., Denisov, E., Damoc, E., Kuehn, A., Makarov, A., and Mann, M. (2014). The Q Exactive HF, a Benchtop mass spectrometer with a pre-filter, high-performance quadrupole and an ultra-high-field Orbitrap analyzer. Mol. Cell. Proteomics *13*, 3698–3708.

67. Callister, S.J., Barry, R.C., Adkins, J.N., Johnson, E.T., Qian, W.J., Webb-Robertson, B.J., Smith, R.D., and Lipton, M.S. (2006). Normalization approaches for removing systematic biases associated with mass spectrometry and label-free proteomics. J. Proteome Res. *5*, 277–286.

OMTM, Volume *17*

Supplemental Information

Optimization of 5′ Untranslated Region of Modified mRNA for Use in Cardiac or Hepatic Ischemic Injury

Nishat Sultana, Yoav Hadas, Mohammad Tofael Kabir Sharkar, Keerat Kaur, Ajit Magadum, Ann Anu Kurian, Nadia Hossain, Bremy Alburquerque, Sakib Ahmed, Elena Chepurko, and Lior Zangi

Supplementary Information

Table of Contents

1) Supplementary Figures

2) Supplementary Table 1

3) Author Contributions

1. Supplementary figures

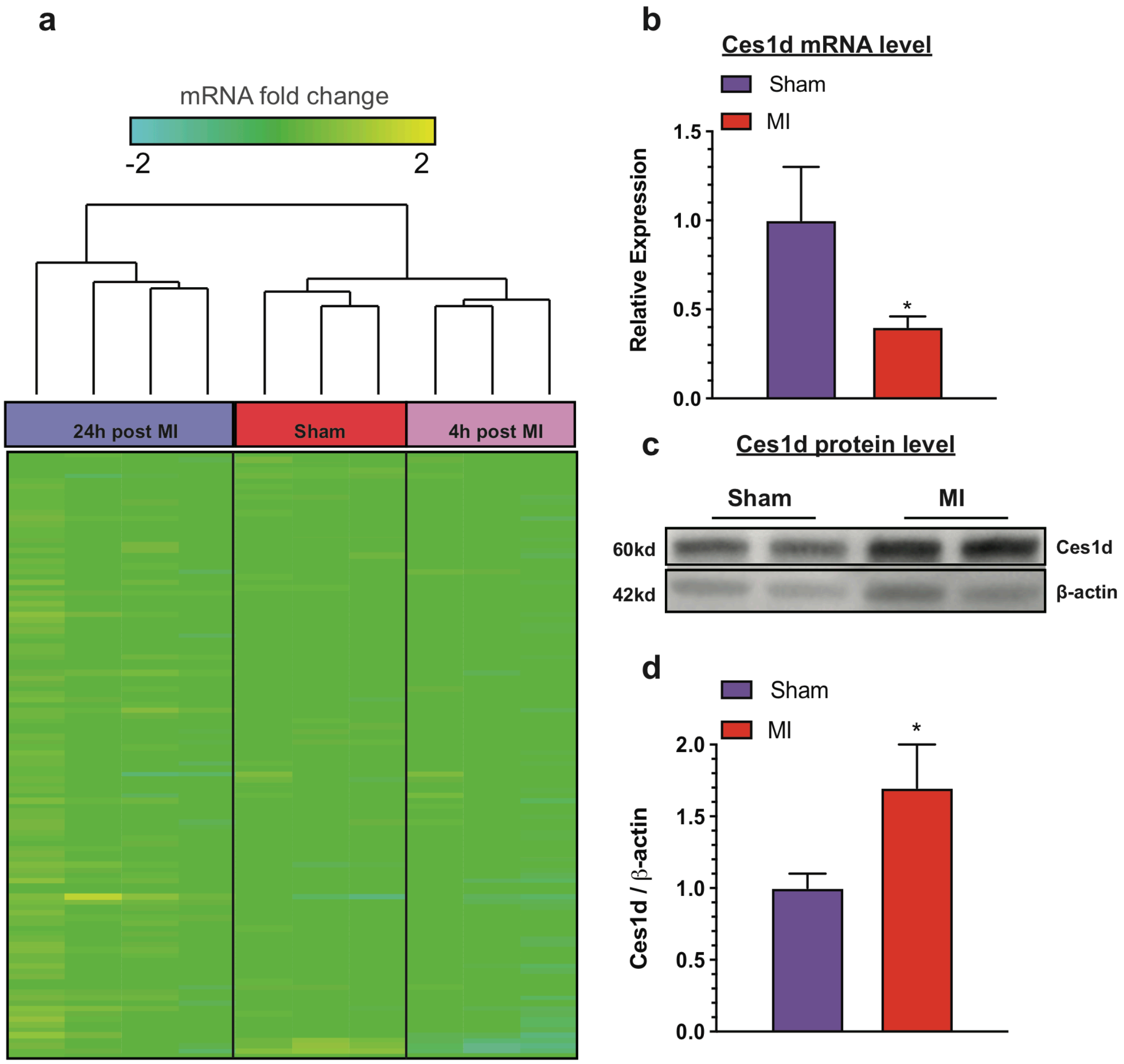

Supplementary Figure 1: RNAseq experimental groups are clusters together in hierarchical clustering dendrogram and Ces1d qPCR and western blot confirm RNAseq and proteomic expression results. a. Hierarchical clustering dendrogram of 14,000 genes that was sequenced for transcriptomic analysis from ischemic or non-ischimic heart tissues (n=10 total, Sham n=3, 4 hours post MI n=3 or 24 hours post MI n=4). **b.** qPCR to evaluate Ces1d expression was done for RNA samples taken 24 hours from hearts undergo Sham or MI surgery (n=3). **c.** Representative image of western blot analysis done with anti Ces1d antibody to evaluate protein samples taken 24 hours from hearts undergo Sham or MI surgery. **d.** Quantification of the experiments shown in c (n=2). Unpaired two-tailed t-test for b&d. *, P<0.05.

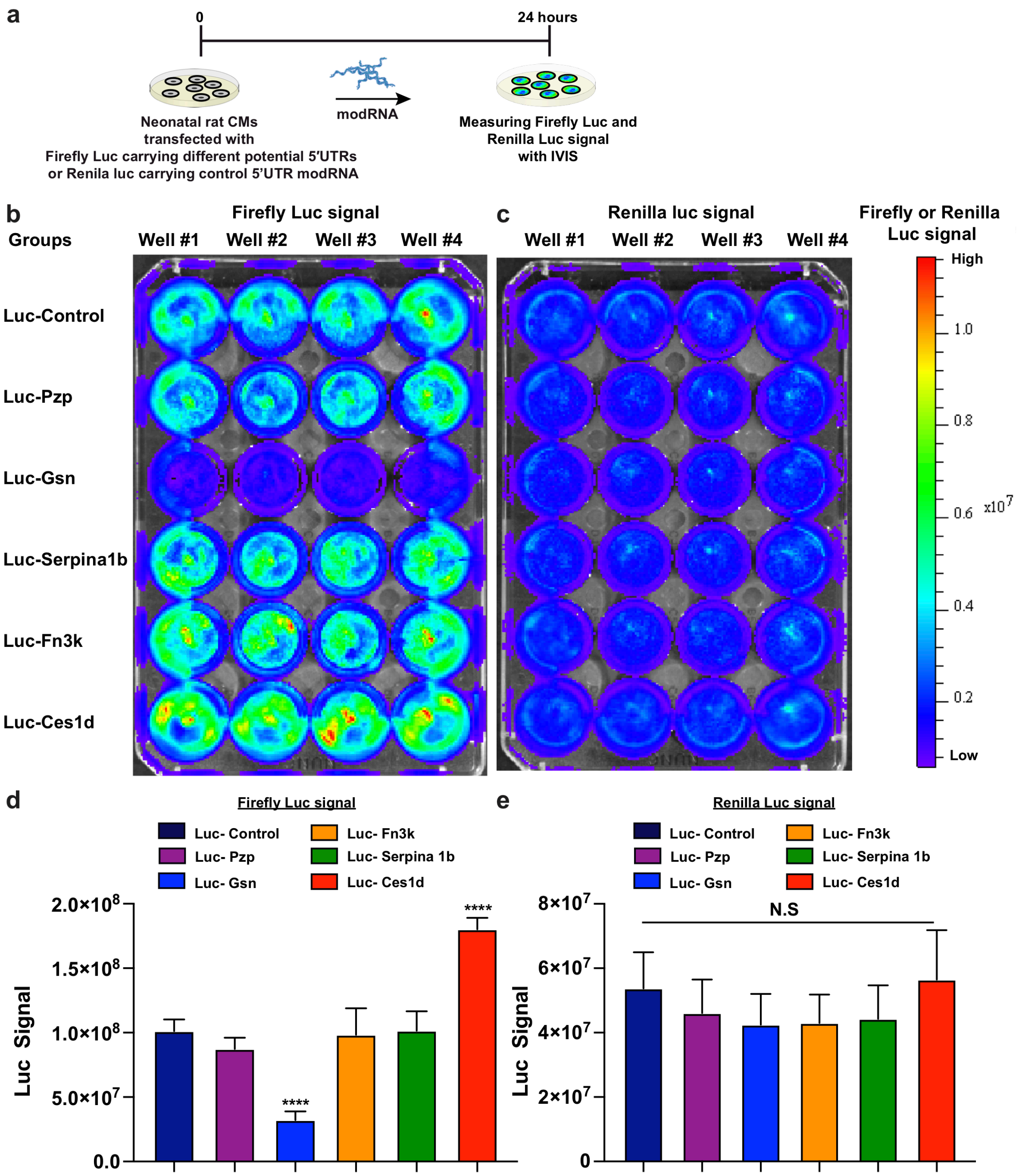

Supplementary Figure 2. Representative image from IVIS analysis 24 hours post transfection with Luc modRNA carrying different potential 5′UTRs and Renilla modRNA (transfection control) in neonatal rat CMs.

a. Experimental plan to evaluate the translation efficiency of different potential 5′UTRs in neonatal rat CMs using Luc modRNA and IVIS analysis.

b&c. Representative IVIS analysis (**b** for luciferase (Luc) signal and **c** for renilla signal) image of the results that was received in the experiment that was described in a. **d&e.** Quantification of the IVIS analysis that describe in b&c, respectively. One-way ANOVA, Tukey's Multiple Comparison Test for d&e.
****, P<0.0001, ***, P<0.001, N.S, Not Significant.

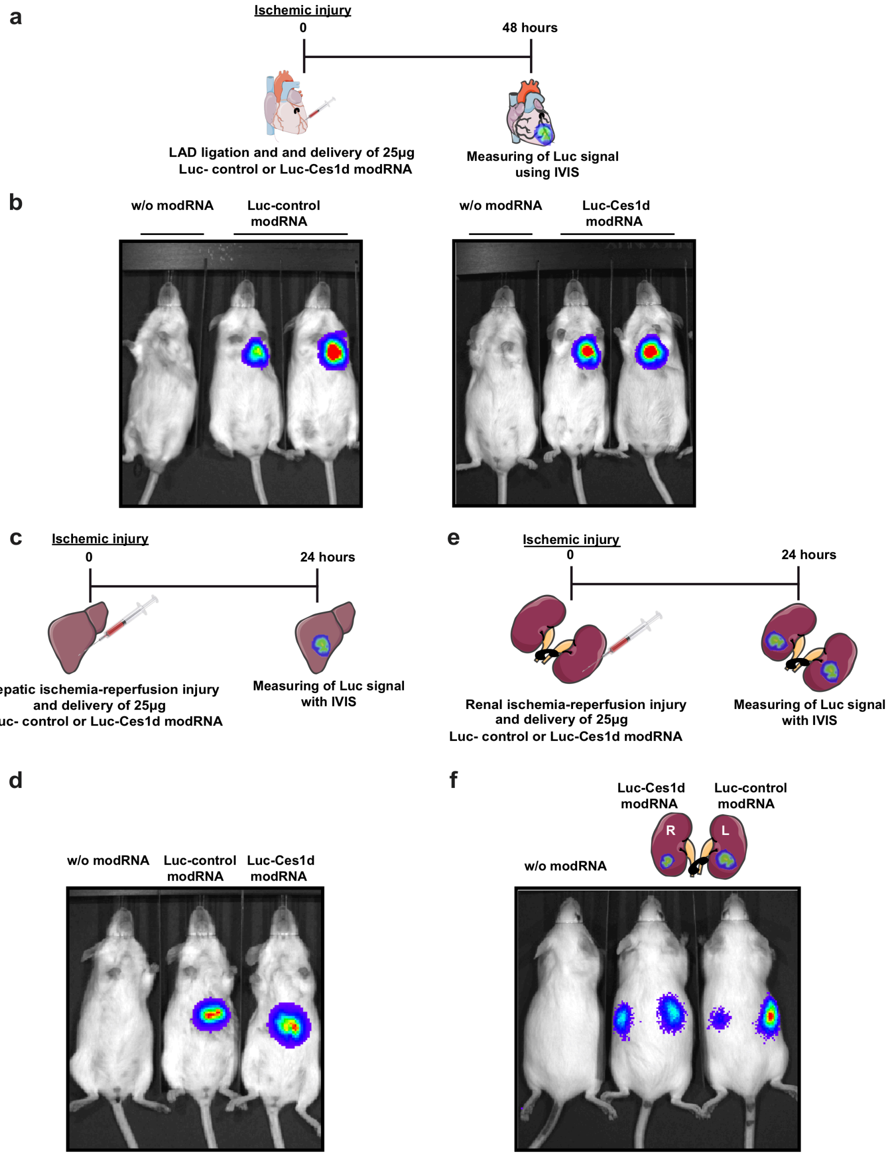

Supplementary Figure 3. Representative images of Luc modRNA translation efficiency when carrying 5′UTR of Ces1d or artificial (control) 5’UTR in ischemic heart, kidney or liver mouse models.

a. Experimental plan to evaluate the translation efficiency of Luc modRNA carrying 5′UTR of Ces1d or artificial (control) 5’UTR in ischemic heart mouse model. **b.** Representative IVIS images of the results that was received in the experiment that was described in a. **c.** Experimental plan to evaluate the translation efficiency of Luc modRNA carrying 5′UTR of Ces1d or artificial (control) 5’UTR in ischemic liver mouse model. **d.** Representative IVIS image of the results that was received in the experiment that was described in c. **e.** Experimental plan to evaluate the translation efficiency of Luc modRNA carrying 5′UTR of Ces1d or artificial (control) 5’UTR in ischemic kidney mouse model. **f.** Representative IVIS image of the results that was received in the experiment that was described in e.

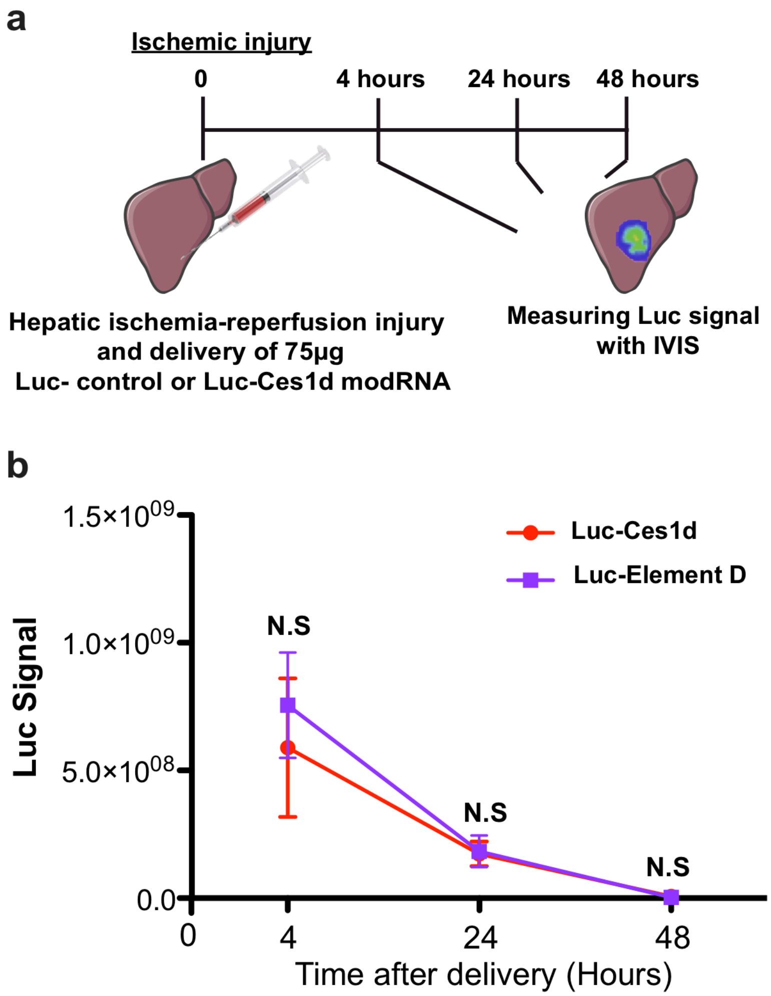

Supplementary Figure 4. Element D in 5'UTR of Ces1d hasn't increased significantly mRNA translation over full length Ces1d 5'UTR in liver ischemic mouse model.

a. Experimental plan to evaluate the translation efficiency of Luc modRNA carrying full length 5′UTR of Ces1d or only Element D in ischemic liver mouse model. **b.** Quantification of the experiments that was described in a (n=4). Two-way ANOVA, Tukey's Multiple Comparison Test. N.S, Not Significant.

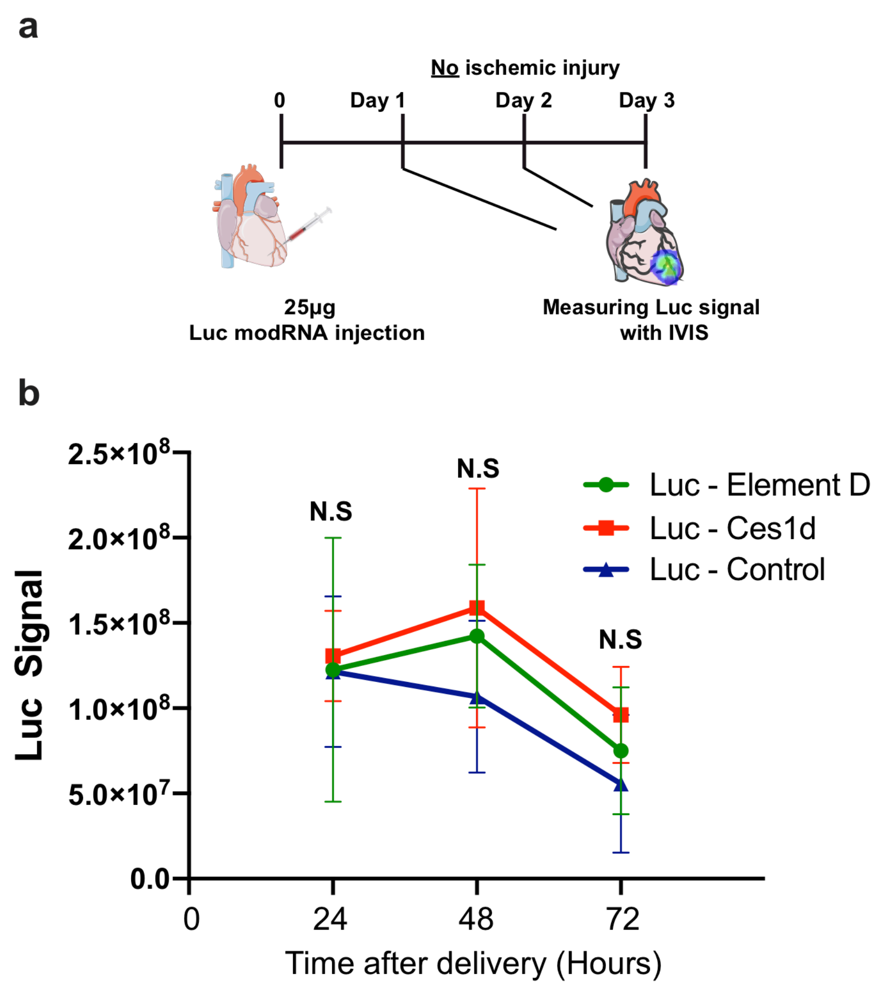

Supplementary Figure 5. Element D in 5'UTR of Ces1d hasn't increased significantly mRNA translation over full length Ces1d or control 5'UTR in heart non-ischemic mouse model.

a. Experimental plan to evaluate the translation efficiency of Luc modRNA carrying full length Ces1d or control 5'UTR in heart non-ischemic mouse model. **b.** Quantification of the experiments that was described in a (n=6). Two-way ANOVA, Tukey's Multiple Comparison Test. N.S, Not Significant.

2. Supplementary Table

Supplementary Table 1: 5'UTR sequence of the 5 genes with the shortest 5'UTR length from the list in Fig. 1f&g.

Gene name	5'UTR Sequence
Gsn	gctagggcgggatgggacggccggttacttaaaggttggg gcgaccaagggtccgcggccgcagcctgggtcctcaccgt cgcc
Pzp	caaggatcagagttcgggggctgagggctcagacgttcttctctgccctc tccacc
Serpina 1b	atatccccttggctcccactgcttaaatacagactaggacagggctctg tctcctcagcctcggtcaccacccagctctgggacagcaagctgaaa
Fn3k	tgcgtcacctgaccgcattctgcacctcaactctcc
Ces1d	aggaggcgggtcccctggtccacaacagaagcattgctaaagcagcagat agctcagagacccacagagcccttgtccttccaca

3. Author Contributions

N.S. designed the study, carried out most of the experiments, analyzed most of the data and wrote the manuscript, Y.H. designed, carried out and analyzed the RNAseq and proteomics experiments and revised the manuscript, M.T.K.S prepared modRNAs and performed experiments, K.K. performed experiments and revised the manuscript, A.M. revised the manuscript, N.H., B.A and S.A., perform experiment and help analyzing the data, E.C. carried out all surgery and modRNA injections in mouse models, L.Z. designed the experiments, analyzed data and wrote the manuscript .
